# Batch-Learning Self-Organizing Map Identifies Horizontal Gene Transfer Candidates and Their Origins in Entire Genomes

**DOI:** 10.3389/fmicb.2020.01486

**Published:** 2020-07-03

**Authors:** Takashi Abe, Yu Akazawa, Atsushi Toyoda, Hironori Niki, Tomoya Baba

**Affiliations:** ^1^Department of Information Engineering, Faculty of Engineering, Niigata University, Niigata, Japan; ^2^Comparative Genomics Laboratory, National Institute of Genetics, Mishima, Japan; ^3^Advanced Genomics Center, National Institute of Genetics, Mishima, Japan; ^4^Microbial Physiology Laboratory, National Institute of Genetics, Mishima, Japan; ^5^Joint Support-Center for Data Science Research, Research Organization of Information and Systems, Tokyo, Japan

**Keywords:** horizontal gene transfer, Antarctic environment, *Sphingomonas* genome, Batch-Learning Self-Organizing Map, oligonucleotide, low-temperature adaptation, amino acid frequency, convergent evolution

## Abstract

Horizontal gene transfer (HGT) has been widely suggested to play a critical role in the environmental adaptation of microbes; however, the number and origin of the genes in microbial genomes obtained through HGT remain unknown as the frequency of detected HGT events is generally underestimated, particularly in the absence of information on donor sequences. As an alternative to phylogeny-based methods that rely on sequence alignments, we have developed an alignment-free clustering method on the basis of an unsupervised neural network “Batch-Learning Self-Organizing Map (BLSOM)” in which sequence fragments are clustered based solely on oligonucleotide similarity without taxonomical information, to detect HGT candidates and their origin in entire genomes. By mapping the microbial genomic sequences on large-scale BLSOMs constructed with nearly all prokaryotic genomes, HGT candidates can be identified, and their origin assigned comprehensively, even for microbial genomes that exhibit high novelty. By focusing on two types of *Alphaproteobacteria*, specifically psychrotolerant *Sphingomonas* strains from an Antarctic lake, we detected HGT candidates using BLSOM and found higher proportions of HGT candidates from organisms belonging to *Betaproteobacteria* in the genomes of these two Antarctic strains compared with those of continental strains. Further, an origin difference was noted in the HGT candidates found in the two Antarctic strains. Although their origins were highly diversified, gene functions related to the cell wall or membrane biogenesis were shared among the HGT candidates. Moreover, analyses of amino acid frequency suggested that housekeeping genes and some HGT candidates of the Antarctic strains exhibited different characteristics to other continental strains. Lys, Ser, Thr, and Val were the amino acids found to be increased in the Antarctic strains, whereas Ala, Arg, Glu, and Leu were decreased. Our findings strongly suggest a low-temperature adaptation process for microbes that may have arisen convergently as an independent evolutionary strategy in each Antarctic strain. Hence, BLSOM analysis could serve as a powerful tool in not only detecting HGT candidates and their origins in entire genomes, but also in providing novel perspectives into the environmental adaptations of microbes.

## Introduction

Although genetic information is generally inherited vertically, i.e., from an organism’s ancestor to its offspring, it can occasionally be transferred horizontally in a process called horizontal gene transfer (HGT) between organisms that do not share an ancestor-offspring relationship (Andam and Gogarten, 2011). HGT was first experimentally demonstrated in *Escherichia coli* in the late 1940s (Tatum and Lederberg, 1947; Soucy et al., 2015), however, the potential evolutionary implications of cross-species HGT was first appreciated only in the late 1980s (Syvanen, 1985, 2012). Since this time, HGT studies have focused primarily on the phylogenetic relationships among organisms, as revealed through similarity searches of nucleotide or amino acid sequences, and were related to taxonomy, the last universal common ancestor, and the Tree of Life (Andam and Gogarten, 2011; Syvanen, 2012; Soucy et al., 2015). Recent innovations in DNA sequencing have led to a massive increase in the amount of genomic and metagenomic data leading to not only an expansion of the interpretations of the Tree of Life through the identification of previously unknown genes, genomic information, or organisms using cultivation-independent genomics approaches, but also the prediction of potentially enormous numbers of HGT cases (Hug et al., 2016; Parks et al., 2017; Schulz et al., 2017; Williams et al., 2017; Castelle and Banfield, 2018).

In terms of its biological significance, HGT plays a dominant role in the evolution and environmental adaptation of an organism (Ochman et al., 2000; Gogarten and Townsend, 2005; Keeling and Palmer, 2008; Darmon and Leach, 2014; Booth et al., 2016). For instance, certain HGTs have been suggested to be associated with adaptation to living in various adverse environments, such as cold (Blanc et al., 2012; Raymond and Kim, 2012; Zeng et al., 2016; Mock et al., 2017), hot and acidic (Schonknecht et al., 2013), or high-salt environments (Harding et al., 2017), as well as environments that induce oxidative stress or ultraviolet radiation stress (Fast et al., 2003; Slamovits and Keeling, 2004), and those featuring the combined stresses of desiccation, oxidation, and osmotic pressure (Eyres et al., 2015). Several bioinformatics and genome-wide studies concerning HGT genes have been conducted, including the estimation of HGT gene frequency (Dagan et al., 2008; Zamani-Dahaj et al., 2016), and HGT gene functional analysis (Jain et al., 1999; Nakamura et al., 2004; Beiko et al., 2005; Choi and Kim, 2007). These studies confirmed the global applicability of HGT and their importance in the evolution of species. Furthermore, they employed approaches based on the phylogenetic relationships that rely on sequence alignments and reference datasets. However, currently predicted HGTs remain limited as HGT is typically identified using phylogeny-based methods that rely on sequence alignments. Additionally, the individual evaluation of HGT genes in each genome analysis remains a challenge to be overcome. Using phylogeny-based methods, an HGT is detected once the phylogenetic location of a query sequence in a tree is determined to not match that of a reference phylogeny. Although this approach is standard, the frequency of HGT events might be underestimated, particularly when information on donor sequences is lacking (Tamames and Moya, 2008).

An alternative approach is the composition-based method, which relies on nucleotide compositional features such as G + C percentage or codon usage (Kanaya et al., 1999, 2001; Garcia-Vallve et al., 2000; Nakamura et al., 2004), and thus does not require sequence alignments. Although G + C percentage has been used as a fundamental phylogenetic characteristic of microbial genomes, it is too simple a parameter for the differentiation of a wide variety of genomes. However, oligonucleotide composition can distinguish between species even when they feature the same G + C percentage, as the oligonucleotide composition varies substantially among genomes and thus constitutes a “genome signature” (Karlin et al., 1998; Mrazek, 2009). Moreover, composition-based methods are generally effective at identifying recently acquired HGT events since ancient HGT genes eventually evolve or ameliorate to become similar to the other genome signatures of host organisms (Douglas and Langille, 2019). However, compositional and phylogenetic methods have different sensitivities as they likely target HGT events of different ages, with the compositional method primarily identifying recent transfers, while phylogenetic methods generally detect older events, although sufficient variation is required to alter tree topology of the region imported by HGT (Tamames and Moya, 2008).

To improve existing compositional methods, we have focused on the oligonucleotide composition in genomic sequences and developed an alignment-free clustering method based on an unsupervised neural network: “Batch-Learning Self-Organizing Map (BLSOM).” Neural networks are a typical machine learning method based on the mathematical model of the neuron circuit; meanwhile the Kohonen’s Self-Organizing Map, which is the basis of BLSOM, was proposed based on the mathematical model of self-organization in the cerebral visual cortex (Kohonen, 1990; Kohonen et al., 1996). In this method, sequence fragments are clustered based solely on the similarity of oligonucleotides and in the absence of taxonomical information (Kanaya et al., 2001; Abe et al., 2003). We have previously optimized the BLSOM method for the phylogenetic classification of genomic sequences obtained from the mixed genomes of environmental microbes (Iwasaki et al., 2013; Nakao et al., 2013; Qiu et al., 2019), and analyzed tetranucleotide frequencies (256-dimensional vectorial data) in 5-kb sequence fragments. Furthermore, by focusing on HGT events in relation to the symbiosis between eukaryotes and prokaryotes, we have successfully detected HGT candidates from diverse bacterial origins in the tsetse fly genome despite their low similarity to currently available genomic sequences (Nakao et al., 2016).

In this study, we applied our BLSOM method to detect HGTs in the genomes of two Antarctic bacteria, *Sphingomonas* sp. HMP6 and HMP9, as well as other *Sphingomonas* strains isolated from other continents. The two Antarctic bacteria are taxonomically closely related and were isolated from the same environment, i.e., an Antarctic moss pillar (Nakai et al., 2012). The genus *Sphingomonas* belongs to the family *Sphingomonadaceae* of the class *Alphaproteobacteria* (Takeuchi et al., 2001) and has been isolated from various environments, including water (Yabuuchi et al., 2001), plants (Eevers et al., 2015), and soil (Huang et al., 2009; Tabata et al., 2011; Wei et al., 2015), as well as in the form of cosmopolitan bacteria in all continents on the planet (Huang et al., 2017). Antarctica is an extreme environment, especially for terrestrial organisms, due to its freezing temperatures, desiccation, oligotrophic environment, and intense ultraviolet radiation (Chown et al., 2015). As a consequence, organisms living in Antarctica have evolved to adjust to their environment and have thrived in unique biospheres, including “moss pillars” in some ultra-oligotrophic lakes in Skarvsnes, East Antarctica (Imura et al., 1999). Here we have sought to elucidate the development of evolutionary adaptations of Antarctic bacteria using genome sequence analysis; and have identified many coding HGT genes in these genomes using phylogeny-based methods. However, many genes remain to be identified. As part of a growing fundamental enquiry into the mechanisms responsible for microbe adaptation in recovering and developing biospheres, it is imperative to determine the proportion of coding HGT genes in their genomes, the origin of these HGT genes, as well as which types of functional HGT genes have become incorporated into a host organism’s genome and their role in the organism’s adaptation to its living environment. This study is a first step in analyzing the genomes of these organisms to elucidate the strategies used to adapt to their living environment. By focusing on the genomes of two Antarctic *Sphingomonas* species, we detected HGTs and sought to unravel the process by which the HGT candidate genes were acquired. BLSOM analysis could serve as a powerful tool for facilitating the detection of HGTs and their origins in entire genomes.

## Materials and Methods

### Isolation and Taxonomic Analysis of Antarctic *Sphingomonas* Strains

We collected Antarctic *Sphingomonas* strains from an Antarctic moss pillar on January 19, 2000 from lake Hotoke-Ike at 69°28’ S and 39°34’ E in Skarvsnes, East Antarctica (Nakai et al., 2012). To isolate the bacteria, the frozen Antarctic moss pillar sample was slowly melted at room temperature, after which the resultant lake water was recovered, diluted aseptically with sterilized water, spread onto BG11-agar plates (Stanier et al., 1971) containing 1% glucose, and incubated at 10°C for several weeks. Single colonies growing on these plates were purified by transferring them onto new plates and incubating for several additional weeks at 10°C. The isolated strains were subsequently grown in liquid BG11 medium with 1% glucose at 10°C and stored at –80°C after the addition of glycerol to reach a final concentration of 25%.

For the 16S rRNA gene sequencing and taxonomic analysis, we performed PCR using the bacterium-specific 27F and the universal 1492R primers (DeLong, 1992) for 16S rRNA gene amplification, following the reaction conditions described by Naganuma et al. (2007). The amplified segments were cloned using a TOPO TA cloning kit (Invitrogen, Carlsbad, CA, United States), and were used to transform *E. coli* TOP10 (Invitrogen). The resultant 16S rRNA gene inserts were sequenced using an ABI 3100 automatic DNA-sequencer (Applied Biosystems, Foster City, CA, United States), and the obtained 16S rRNA nucleotide sequences were analyzed phylogenetically using MEGA 6.0 (Tamura et al., 2013) and the maximum-likelihood method based on the Tamura-Nei model (Tamura and Nei, 1993).

Bacterial growth at different temperatures (4, 10, 15, 20, 25, and 30°C) was investigated in liquid BG11 medium containing 1% glucose for up to 8 weeks. The optimal growth was also evaluated at 15°C in these liquid media: BG11, BG11 including 1% glucose, and R2A (Reasoner and Geldreich, 1985).

### Genome Sequencing and Bioinformatic Analysis of Genomic Data

The Antarctic *Sphingomonas* strains were grown in 5 mL of R2A medium at 15°C with shaking (150 rpm) until the OD_600_ reached ∼1.0. Cells were harvested by centrifugation at 8000 × *g* for 5 min, and the genomic DNA was isolated from the pelleted cells by using a Qiagen Genomic-tip 100/G column (Qiagen, Hilden, Germany) according to the manufacturer’s instructions. The genome sequences of the *Sphingomonas* strains were determined using Illumina paired-end sequencing (Illumina Inc., San Diego, CA, United States) and single-molecule real-time DNA sequencing (Pacific Biosciences, Menlo Park, CA, United States). First, whole-genome sequencing data were generated on a PacBio RSII platform and assembled using the HGAP2 assembler (Pacific Biosciences). The assembled genome sequences were corrected using the Quiver software (Pacific Biosciences). Next, Illumina paired-end libraries were constructed and sequenced using an Illumina MiSeq, and the Illumina sequence data were mapped against the genome sequences by BWA v0.7.7 with the option “-M” (Li and Durbin, 2009) for error correction.

The refined genomic data were annotated using various software tools. Genes were identified by MiGAP (Sugawara et al., 2009) and MetaGeneAnnotator (Noguchi et al., 2008), while GLIMMER (Delcher et al., 1999) predicted the coding sequences (CDSs), after which manual curation was performed. For example, in the case of the predicted CDS, wherein the start position of the chromosomal replication initiator protein DnaA was at a “GTG” codon following an “ATG,” the CDS start position was manually curated at the “ATG.” The predicted CDSs were translated and used to search for genes in other organisms in the non-redundant databases provided by the National Center for Biotechnology Information (NCBI_nr) in Feb. 2016, UniProt release 2016_2 (The UniProt, 2018) and STRING v9.1 (Szklarczyk et al., 2019), through BLASTp (Altschul et al., 1990). Top hits with an *E*-value threshold of 1e-5 or lower were used to describe each of the predicted proteins encoded by the identified genes. Genes encoding tRNAs were manually annotated using the tRNADB-CE database group’s strategy (Abe et al., 2011). This tRNA prediction was performed by running three prediction programs used for tRNA gene search, namely tRNAscan-SE v2.0 (Lowe and Eddy, 1997), ARAGORN v1.2.38 (Laslett and Canback, 2004), and tRNAfinder v1.4 (Kinouchi and Kurokawa, 2006). The tRNA genes obtained were defined as reliable without further checks. Next, the residual discordant cases were checked manually based on tRNA secondary structure. Non-coding genes and miscellaneous features were predicted using RNAmmer (Lagesen et al., 2007).

To assign taxonomic information using sequence similarity searches for the strains HMP6 and HMP9, we performed BLASTp against NCBI_nr in February 2016. We examined the taxonomic information of sequences showing a best-hit with an *E*-value of 1e-5 or less, with a similarity to both sequence identity and coverage of 70% or more as the threshold value.

For the comparative analysis based on an orthologous gene group, orthologous genes were extracted by surveying bidirectional best-hit (BBH) relationships among *Sphingomonas* strains using BLASTp with an *E*-value threshold of 1e-5.

To ascertain the types of function acquired by HGT candidates, we compared their functional classification based on the Clusters of Orthologous Groups of Proteins (COG), a gene ortholog database provided by NCBI (Galperin et al., 2015). The functional categories in COG were determined through BLASTp analysis performed using the amino acid sequences registered in COG released in 2014. The COG functional categories of the genes were then obtained using the top-hit with an *E*-value of 1e-5 or less as the threshold value.

### Batch-Learning Self-Organizing Map

Kohonen’s Self-Organizing Map (SOM), an unsupervised neural network algorithm, is a powerful tool for clustering and visualizing high-dimensional complex data on a two-dimensional map (Kohonen, 1990; Kohonen et al., 1996). We modified the conventional SOM for genome informatics on the basis of batch-learning to develop BLSOM, aiming to make the learning process and the resulting map independent of the order of data input (Kanaya et al., 2001; Abe et al., 2003). The newly developed SOM is suitable for high-performance parallel computing and therefore for big data analysis.

The initial weight vectors were defined using principal component analysis (PCA) based on the variance-covariance matrix, rather than by using random values, as PCA can classify gene sequences into groups of recognized biological categories. The weight vectors (**w***_*ij*_*) were arranged in a two-dimensional lattice denoted by *i* (=0, 1,.., *I*-1) and *j* (=0, 1,.., *J*-1) (where *I* and *J* represent the size of two-dimensional lattice points in first and second dimensions, respectively) and were set and updated as described previously (Kanaya et al., 2001; Abe et al., 2003). A BLSOM program suitable for PC cluster systems is available on our website^1^.

### Detecting HGT Candidates in Microbial Genomes and Predicting Their Origin

**FIGURE 1:**
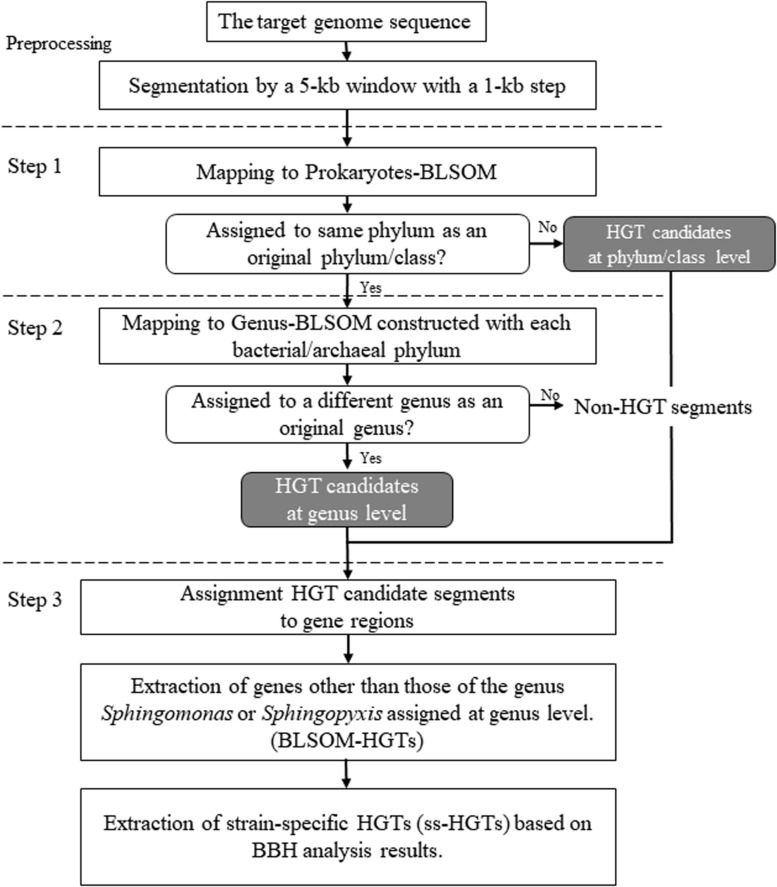
Workflow for detecting HGT candidates in the microbial genomes using BLSOM.

An overview of the proposed workflow is presented in Figure 1. To create sequence datasets for BLSOM analysis, bacterial nucleotide and draft genome sequences were obtained from GenBank^2^ and the whole-genome shotgun division in DDBJ^3^, respectively, in January 2016. The registered sequence length of 5 kb or more was extracted and grouped by species. Here, allogeneic strains were grouped as the same species, and plasmid sequences were excluded. When the total number of nucleotides grouped by each species reached 10 kb or more, these sequences were aggregated per genus and concatenated by adding ten undetermined nucleotides (Ns) between the sequences, resulting in a single concatenated sequence per genus. When the number of Ns in a sequence exceeded 10% of the window size, the sequence was omitted from analysis, while when it was less than 10%, the oligonucleotide frequencies were normalized to the length without Ns and included in the analysis. The genus names and the number of 5 kb segments, which were used as sequence datasets, are shown in Supplementary Table S1.

In this workflow, two types of large-scale BLSOMs, namely Prokaryotes-BLSOM and Genus-BLSOM, were constructed to identify HGT candidates in microbial genomes. These BLSOMs were constructed using a degenerate tetranucleotide composition totaling 5,200,000 5-kb sequences from 1,217 genera, of which at least 10 kb of sequence was available from DDBJ/ENA/GenBank. The degenerate tetranucleotide composition refers to the composition of degenerate sets in which a pair of complementary tetranucleotides was added (e.g., ATGC and GCAT). Hence, our previous BLSOM analysis of a wide range of species revealed that sequences from a single genome often form a vertically mirror-symmetrical cluster (Abe et al., 2003), e.g., according to the replicational direction of the genomic fragments.

During preprocessing, a 5-kb window featuring a 1-kb step was used (“Preprocessing” in Figure 1), which was mapped to Prokaryotes-BLSOM by identifying the lattice point with the minimum Euclidian distances of the degenerate tetranucleotide composition between the genomic segment and the lattice points (“Step 1” in Figure 1). For every lattice point at which microbial genomic segments were mapped into phylum/class territories, the most abundant phylum/class was identified, and the mapped microbial genomic segments were assigned to this phylum/class by BLSOM. To identify the taxonomic origin of the microbial genomic segments that were mapped to the prokaryotic territories on Prokaryotes-BLSOM, the segments were mapped on Genus-BLSOM (“Step 2” in Figure 1). The microbial genomic segments were mapped in a similar stepwise manner on Genus-BLSOMs constructed using sequences from more detailed taxonomic categories (e.g., genera).

To assign the microbial genomic segments to specific gene regions, the taxonomic category of a gene was assigned the taxonomic category of the microbial genomic fragment that covered the entire gene region and whose start position was the closest (“Step 3” in Figure 1). Next, the genes other than those of the genus *Sphingomonas* or *Sphingopyxis* assigned by Genus-BLSOM were defined as HGT candidates (abbreviated as BLSOM-HGTs). Furthermore, from these BLSOM-HGTs, by performing BBH analysis among the *Sphingomonas* strains, only those genes that showed similarity only to themselves or whose BBH was not established were defined as strain-specific HGT candidates (abbreviated as ss-HGTs).

The HGT candidate detection of private genomic sequence segments, such as that of new species, against Prokaryotes- and Genus-BLSOM can be performed by applying the phylogenetic estimation software for metagenomic sequences that we have previously developed. The software and BLSOM data are available on our website^4^.

### Amino Acid Frequency Analysis for Investigating Low-Temperature Adaptation in Antarctic *Sphingomonas* Strains

Cold-adapted enzymes feature a low arginine content and a low Arg/(Arg + Lys) ratio, as these are well-suited for their low-temperature activity and thermal instability (Russell, 2000). We sought to further elucidate the role of the arginine content to comprehensively investigate low-temperature adaptation in the Antarctic environment by comparing the amino acid frequencies in proteins encoded by genes (4,280 and 1,712 genes from continental and Antarctic strains, respectively), for which BBH relationships were established in eight strains, including a *Zymomonas* strain. By following this selection strategy, a *t*-test was performed for each amino acid. Among the amino acids that showed significant differences, Lys, Ser, Thr, and Val were selected as the amino acids with frequencies higher in Antarctic strains than in continental strains, whereas Ala, Arg, Glu, and Leu were selected as the amino acids with frequencies lower in Antarctic strains than in continental strains. The low-temperature relation ratio for Antarctic *Sphingomonas* strains based on the eight selected amino acids is as follows:

(1) $$\frac{A\,l\,a+A\,r\,g+G\,l\,u+L\,e\,u}{A\,l\,a+A\,r\,g+G\,l\,u+L\,e\,u+L\,y\,s+S\,e\,r+T\,h\,r+V\,a\,l}$$

This ratio is based on the ratio of amino acids that are decreased in the Antarctic environment, and thus the value is low in proteins encoded by the genes of Antarctic strains.

### Statistical Analysis

Considering that the statistical analyzes used differed depending on the normality and equal variance of the two samples compared, Kolmogorov–Smirnov test was first performed to test normality. If the two compared samples were normally distributed, *F*-test for equality of variance was performed. We also performed the two-sample test for assessing differences between two population means to compare amino acid frequencies. Student’s *t*-test was used when normal distribution and equality of variance were observed, Welch’s *t*-test was applied when normal distribution was observed without equality of variance, and Wilcoxon rank-sum test was used if normality was not observed. Finally, the *p*-value obtained was adjusted by Benjamini-Hochberg procedure. All statistical tests were performed in R package (R Core Team, 2019).

## Results and Discussion

### *Sphingomonas* Strains HMP6 and HMP9 Are Closely Related to Psychrotolerant Bacteria

**FIGURE 2:**
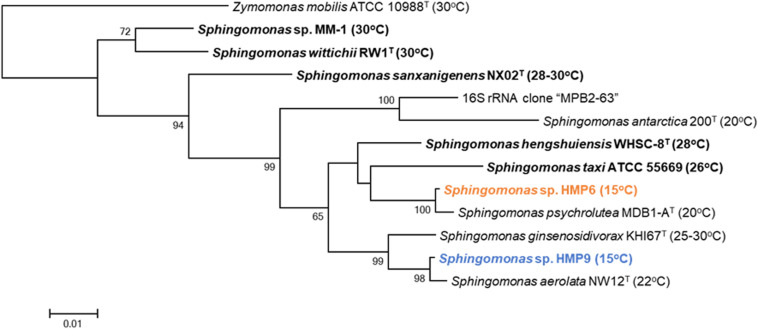
Phylogenetic tree of the 12 *Sphingomonas* strains based on 16S rRNA sequence data constructed using the maximum-likelihood method under the Tamura-Nei model. Species names in bold are genome-sequenced strains. The numbers before the branching points correspond to bootstrap percentage values for 1000 replicates, whereas the superscript “T” indicates type strains. Values inside parentheses are the temperatures for optimum growth. Orange and blue text represents the Antarctic HMP6 and HMP9 strains, respectively.

Comparative 16S rRNA sequence analysis of the two *Sphingomonas* strains HMP6 and HMP9 revealed a 95.5% similarity. Results from our previous study analyzing the microflora of the Antarctic moss pillar, using 16S rRNA clone libraries, suggested the existence of *Sphingomonas* species as one 16S rRNA clone, “MPB2-63” (Nakai et al., 2012). MPB2-63 showed 94.4% sequence similarity to both HMP6 and HMP9, and the highest similarity to MPB2-63 based on the current 16S rRNA database (accessed on Jan. 2020; O’Leary et al., 2016)^5^ was found in the case of *Sphingomonas antarctica* 200 (Huang et al., 2017). A phylogenetic tree obtained using the maximum-likelihood method in MEGA 6.0 (Tamura et al., 2013) software is presented in Figure 2, which shows the phylogenetic relationships based on the 16S rRNA sequence data of 12 *Sphingomonas* strains and the clone MPB2-63 (Nakai et al., 2012). *Zymomonas mobilis* ATCC 10988 (Skotnicki et al., 1981), a genome-sequenced type strain (Pappas et al., 2011), was used as the tree outgroup. The multiple sequence alignment in the FASTA format and the phylogenetic tree in the Newick format in Figure 2 are presented as Supplementary Materials S1, S2, respectively. The publicly available isolated strains that represent the closest relatives to the Antarctic *Sphingomonas* strains HMP6 and HMP9 and the clone MPB2-63, are *Sphingomonas psychrolutea* MDB1-A (Liu et al., 2015), *Sphingomonas aerolata* NW12 (Busse et al., 2003), and *S. antarctica* 200 (Huang et al., 2017), respectively. These *Sphingomonas* strains are reported as psychrotolerant bacteria isolated from low-temperature environments, such as glacial ice in Tibet (Liu et al., 2015), airborne in United Kingdom (Busse et al., 2003), and tundra soil in Antarctica (Huang et al., 2017). The *Sphingomonas* strains HMP6 and HMP9 were found to grow at 4–25°C, with an optimal growth at 15°C, suggesting that they exhibit the growth feature of psychrotolerant bacteria. We also analyzed the phylogenetic relationships based on the 16S rRNA sequence data with an additional 24 completely sequenced *Sphingomonas* strains (Supplementary Figure S1). The multiple sequence alignment in the FASTA format and the phylogenetic tree in the Newick format in this figure are presented as Supplementary Materials S3, S4, respectively. The Antarctic *Sphingomonas* strains, namely HMP6, HMP9, and *S. antarctica* 200 (Huang et al., 2017) and the clone MPB2-63 belong to three independent groups.

The optimal growth temperature of each *Sphingomonas* species has not yet been comprehensively reported, and limited information is presented in Supplementary Tables S2, S3. Depending on the optimal growth temperature, different culture media are used (Supplementary Table S2). For example, *Sphingomonas* sp. MM-1, *Sphingomonas wittichii* RW1, *Sphingomonas sanxanigenens* NX02, and *Sphingomonas hengshuiensis* WHSC-8, which showed growth at ∼30°C, used relatively nutrient rich media, such as 1/3 LB medium, Difco nutrient broth, NK medium, and NB medium; meanwhile, psychrotolerant *S. aerolata* NW12, *S. antarctica* 200, *Sphingomonas* sp. HMP6, and *Sphingomonas* sp. HMP9 used the nutrient poor R2A medium. Moreover, *Sphingomonas ginsenosidivorax* KHI67, which showed intermediate growth at temperatures between 25 and 30°C, also used the R2A medium. Although comparison of the optimal growth temperatures for all *Sphingomonas* species using the same culture medium is necessary, here we only evaluated them roughly, while focusing on the *Sphingomonas* strains with well-reported genomic features and their growing temperatures, as well as those of the newly sequenced strains as listed in Figure 2.

### Genome Structures of the Antarctic Strains Are Not Conserved in Continental Strains

The summary genome information of the Antarctic *Sphingomonas* strains HMP6 and HMP9, as well as the other genome-sequenced *Sphingomonas* strains, are presented in Table 1. The detailed gene annotation data for these genomes are shown in Supplementary Tables S4–S6. In addition to a circular chromosome, HMP9 carries a plasmid (designated as pHMP9) ∼43 kb in size, whereas HMP6 does not carry a plasmid. The G + C percentage of pHMP9 is 63%, which is lower than that of the HMP9 genome (66%). The plasmid contains 46 predicted genes, one of which, pHMP9_0003, likely encodes the replication protein RepB (RefSeq: WP_010165325), which shows a high level of conservation with MC45_RS18375 on *Sphingomonas taxi* ATCC 55669 plasmid STP2 (Eevers et al., 2015) and with G432_21330 on *Sphingomonas* sp. MM-1 plasmid pISP1 and G432_RS21815 on plasmid pISP4 (Wu et al., 2017). A phylogenetic relationship for the RepB genes among these plasmids is shown in Supplementary Figure S2, wherein multiple sequence alignment in the FASTA format and the phylogenetic tree in the Newick format are shown in Supplementary Material S5, S6, respectively. Besides pHMP9_0003, certain other genes encoded on pHMP9 are conserved in *S. taxi* ATCC 55669 plasmid STP2 (Eevers et al., 2015) and in *Sphingomonas* sp. MM-1 plasmids pISP0, pISP1, pISP2, pISP3, and pISP4 (Wu et al., 2017), suggesting that pHMP9 represents one of the typical plasmid structures harbored in the genus *Sphingomonas*.

**TABLE 1:** Genome information and number of HGT candidate genes.

Species	S. sp. HMP6	S. sp. HMP9	*S. taxi* ATCC 55669	*S. hengshuiensis* WHSC-8	*S. sanxanigenens* NX02	*S. wittichii* RW1	S. sp. MM-1
#ACC	AP022672	AP022673	CP009571	CP010836	CP006644	CP000699	CP004036
Genome size (base)	3,637,071	3,960,455	3,859,099	5,191,536	6,205,897	5,382,261	4,054,833
Growth temperature (°C)	15	15	26	28	28	30	30
#CDS	3,535	3,586	3,253	4,214	5,855	4,850	3,801
GC%	64.6	65.5	68.0	66.7	66.8	68.4	67.2
#tRNA	48	56	53	48	58	48	55
#rRNA(5S, 16S, 23S)	6	12	9	6	9	6	6
#BLSOM-HGTs*	778	1,112	475	559	1,484	425	602
#ss-HGTs*	469	724	299	398	1,168	316	419
#ss-HGTs assigned to Alphaproteobacteria*	307	275	191	281	1,050	309	374
#ss-HGTs assigned to Betaproteobacteria*	142	394	94	101	101	3	17
#ss-HGTs assigned to other phylum*	20	55	14	16	17	4	28
PiasmidID	–	pHMP9	STP1/SPT2	WHSC-8 piasmid	pNX02	pSWIT01/pSWIT02	pISP0/pISPl/pISP2/pISP3/pISP4
#ACC	–	AP022674	CP009572/CP009573	CP010837	CP011450	CP000700/CP000701	CP004037/CP004038/CP004039/CP004040/CP004041
Piasmid size (base)	–	42,963	163,311/39,492	36,853	374,401	310,228/222,757	275,840/172,140/53,841/43,776/33,183
#CDS	–	46	108/40	33	356	285/210	251/174/51/44/39
GC%	–	62.9	66.7/62.3	62.6	64.6	64.1/61.2	63.5/62.5/62.9/63.3/63.0

If the genome has multiple plasmids, each plasmid is separated by a slash. *The number of CDSs was indicated.

The Antarctic *Sphingomonas* strains HMP6 and HMP9 feature small genome sizes (<4 Mb) and lower G + C percentages (<66%) than those of the other *Sphingomonas* strains and harbor a similar number of protein-coding genes. However, a twofold difference exists in the number of their rRNA genes (Table 1): HMP6 carries two sets of rRNA operons, whereas HMP9 carries four. Previous genomic studies reported that *Sphingomonas* strains in this genus harbor two or three sets of rRNA operons in their genomes (Miller et al., 2010; Tabata et al., 2013; Eevers et al., 2015; Wu et al., 2017). However, from our complete genome sequence data, the *Sphingomonas* strains appear to harbor one to five sets of rRNA operons in their genomes, the minimum of which are found in *Sphingomonas indica* Dd16, *Sphingomonas ginsengisoli* KACC 16858, *Sphingomonas* sp. AE3, *Sphingomonas* sp. YZ-8, *Sphingomonas* sp. XS-10, and *Sphingomonas* sp. HKS19, while the maximum is in *Sphingomonas* sp. IC081 (Supplementary Figure S1 and Supplementary Table S7). The number of rRNA operons in bacterial genomes was suggested to predict the growth rate and growth efficiency in response to resource availability (Roller et al., 2016). Although we did not observe a markedly enhanced growth rate in HMP9 relative to that of HMP6, the growth conditions we examined were only roughly detected and were limited to the liquid media of BG11, BG11 including 1% glucose, and R2A. To better evaluate the relationship between growth and the number of rRNA operons in HMP6 or HMP9, it will be necessary to continuously measure the growth rate and growth efficiency under experimentally controlled conditions in which several nutrients or temperatures are tested. In terms of the numbers of tRNA genes in *Sphingomonas* genomes, HMP6 harbors 48 genes, equal to that in *Sphingomonas hengshuiensis* WHSC-8 and *S. wittichii* RW1, which both carry two sets of rRNA operons. In contrast, HMP9 harbors 56 tRNA genes, similar to *S. taxi* ATCC 55669 and *S. sanxanigenens* NX02, which carry three sets of rRNA operons. In the same survey of complete genome sequence data, *Sphingomonas* strains were found to contain 44—64 tRNA genes in their genomes, of which the minimum 44 is in *Sphingomonas* sp. YZ-8, whereas the maximum 64 are in *Sphingomonas* sp. IC081 (Supplementary Figure S1). These *Sphingomonas* strains present similar “linked rRNA operon structures” (Brewer et al., 2020), consisting of 16S rRNA, Ile-tRNA, Ala-tRNA, 23S rRNA, 5S rRNA, and Met-tRNA. HMP9 includes two additional rRNA operon structures as compared with HMP6, and thus six additional tRNA genes are present in the HMP9 genome.

Although the *Sphingomonas* strains HMP6 and HMP9 did not form a clade (Figure 2 and Supplementary Figure S1), they did exhibits features of psychrotolerant bacteria. This suggests that the adaptation to Antarctic’s low-temperature environment may be achieved via convergent adaptation (independent evolution of similar traits). Furthermore, the genomes of the strains HMP6 and HMP9 were smaller and had lower GC content than those of the other *Sphingomonas* strains (Table 1). According to gene annotation for predicting CDSs by using BLASTp against the NCBI_nr database, only ∼65% of the CDSs of both HMP6 and HMP9 strains were found to be similar to the genes in the genus *Sphingomonas*; ∼10% of the CDSs were similar to the genes in other genera and >25% remained as “no-hits.” These results suggest that the genome structures of HMP6 and HMP9 consist of a large number of genes that do not show conservation with genes in the genus *Sphingomonas*, thereby implying that these strains carry horizontally transferred genes. To test this hypothesis, we used an alignment-free HGT-detection method.

### HGTs of HMP6 and HMP9 Have Different Origins

**FIGURE 3:**
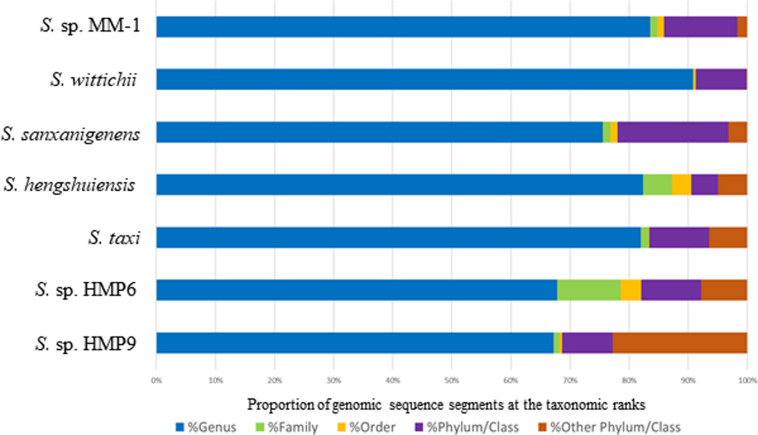
Proportion of genomic sequence segments in the *Sphingomonas* genome-sequenced strains assigned according to taxonomic rank. The horizontal bars represent proportion of genomic segments assigned to each taxonomic rank by BLSOM with taxonomic ranks color-coded: genus (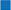), family (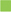), order (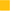), phylum/class (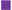), and other phylum/class (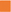).

We used BLSOM to detect HGT candidates and their origins in two Antarctic *Sphingomonas* strains, HMP6 and HMP9 (hereafter, Antarctic strains), and in five strains isolated from continents other than Antarctica with their entire genomes previously decoded (hereafter, continental strains). Figure 3 shows the proportions of the genomic sequence segments of each strain assigned to each taxonomic rank by BLSOM. Firstly, among the continental strains in the genus *Sphingomonas*, *S. wittichii* featured the highest proportion (90.8%), whereas *S. sanxanigenens* had the lowest (75.5%), with the average proportion being 82.8%. Conversely, between the Antarctic strains, the assigned proportions were 67.8% for HMP6 and 67.2% for HMP9. Thus, the proportions assigned to the genus *Sphingomonas* tended to be lower in the Antarctic than in the continental strains. Next, at the *Sphingomonadaceae* family level, the highest proportion was 90.9% for *S. wittichii*, and the lowest was 76.8% for *S. sanxanigenens*, with the average at 84.6%, whereas the proportions were 78.5 and 68.2% for HMP6 and HMP9, respectively. Lastly, for the proportion of genomic sequence segments assigned to the class *Alphaproteobacteria* of the same phylum/class, the highest was 99.8% in *S. wittichii* and the lowest was 93.5% in *S. taxi*, with an average of 96.7% among the continental strains; by comparison, HMP6 featured a high proportion (92.2%) similar to the continental strains, whereas HMP9 was 77.2%, which was lower than those for HMP6 and the continental strains. The remaining 22.8% was assigned to other phylum/classes. This suggests that the Antarctic strains have acquired a higher proportion of HGT candidates than the continental strains, which further supports that the Antarctic strain HMP6 has acquired most of the HGTs from the same family or phylum/class. In contrast, HMP9 contains a higher proportion of HGT candidates acquired from another phylum/class than from either *S. taxi*, a closely related species in the phylogenetic tree, or HMP6, which was isolated from the same Antarctic environment. Thus, HGTs of HMP9 have origins that differ from those of HMP6 and the continental strains.

To determine whether our proposed BLSOM analysis is applicable to draft genomes, we applied BLSOM to detect HGT candidates for the genome sequence segments of *S. ginsenosidivorax* KHI67 and *S. aerolata* NW12, whose draft genomes were decoded in type strains phylogenetically closely related to the Antarctic strain HMP9 (Supplementary Figure S3). The Antarctic strain HMP9 and its phylogenetically closely related strains may have a higher proportion derived from a phylum that differs from that of the other *Sphingomonas* strains, suggesting a tendency to acquire genes from another phylum (Figure 3 and Supplementary Figure S3). In addition, taxonomic assignment was performed on the *Sphingomonas* plasmid genomes (Supplementary Figure S4). pHMP9, as well as the plasmids of other *Sphingomonas* strains, was predicted to be derived from the class *Alphaproteobacteria*, suggesting that it is the common ancestral plasmid of other *Sphingomonas* strains, which is similar to the phylogenetic tree for plasmid RepB gene (Supplementary Figure S2).

**FIGURE 4:**
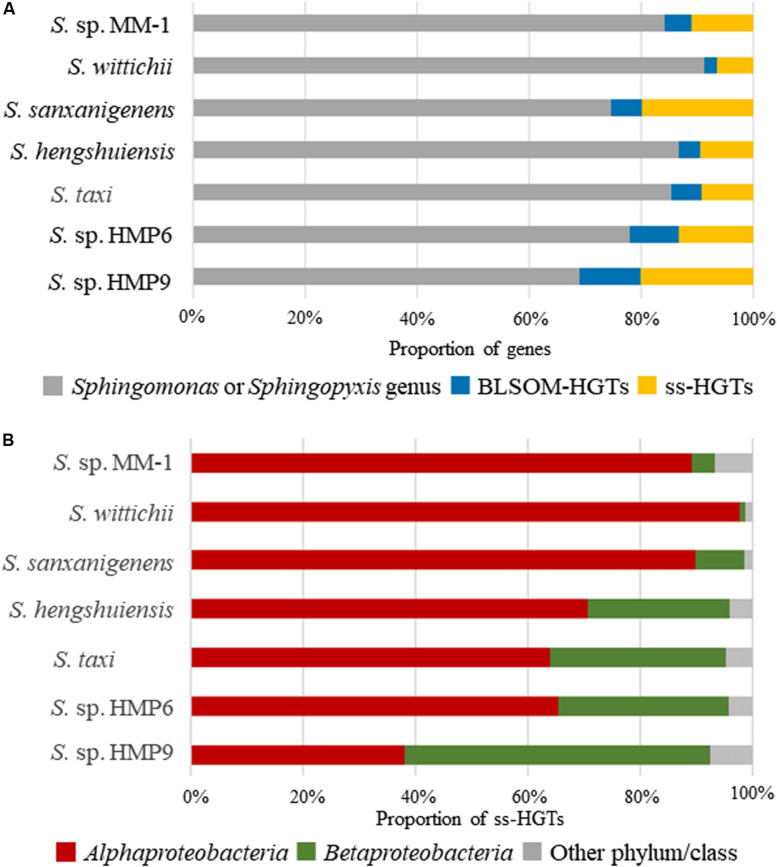
Proportion of HGT candidate genes in the *Sphingomonas* strains assigned by BLSOM and BBH. **(A)** Proportion of HGT candidate genes. Gray (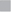): proportion of genes assigned to *Sphingomonas* or *Sphingopyxis* genus; blue (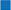): BLSOM-HGTs; orange (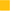): strain-specific HGT candidate genes (ss-HGTs). **(B)** Proportion of ss-HGTs at phylum/class level through BBH analysis from BLSOM-HGTs. Brown (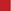): *Alphaproteobacteria*; dark green (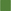): *Betaproteobacteria*; gray (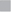): other phylum/class.

Next, we assigned the origin of each gene based on the taxonomic prediction results for genomic sequence segments obtained using BLSOM (Figure 4A). Here, we defined genes other than those assigned to *Sphingomonas* or *Sphingopyxis* by BLSOM as HGT candidates (BLSOM-HGTs). Table 1 shows the number of BLSOM-HGTs. Among the continental strains, *S. sanxanigenens* featured the highest proportion of BLSOM-HGTs (25.4%), whereas *S. wittichii* was the lowest (8.8%), with the average at 15.6%. In comparison, the proportions for HMP6 and HMP9 were 22.0 and 31.0%, respectively. Similar to the prediction results for genomic sequence segments, the prevalence of HGT candidates was higher in the Antarctic than in the continental strains.

To clarify the role of HGT candidates in Antarctic adaptation, we extracted ss-HGT candidates from the BLSOM-HGTs. Here, by using the results of BBH analysis of the Antarctic and continental strains, we defined “ss-HGTs” as genes forming a BBH group only with themselves, or as genes not forming a BBH group. In the Antarctic strains, the proportion of ss-HGTs to BLSOM-HGTs was as high as ∼63%. The ss-HGTs were then comprehensively analyzed as HGT candidates that are highly likely to be involved in Antarctic environmental adaptation. The origin of the ss-HGTs was examined at the phylum/class level as shown in Figure 4B. The continental strains and HMP6 were most frequently assigned to the class *Alphaproteobacteria*, in which the genus *Sphingomonas* belongs, and their next most frequently assigned origin was from the class *Betaproteobacteria*. Conversely, in HMP9, the proportion of ss-HGTs derived from the class *Betaproteobacteria* was the highest, accounting for more than half of the total ss-HGTs. This suggests that HMP9 obtained more HGT candidates from more distantly related bacteria than *S. taxi* and HMP6, which are phylogenetically related. We further examined the origin of the ss-HGTs derived from the class *Alphaproteobacteria* at the family level (Supplementary Figure S5A) and found that the proportion derived from the family *Bradyrhizobiaceae* differed among the strains, whereas that from the family *Erythrobacteraceae* was higher in HMP6 (24.1%) than in HMP9 (4.7%). Moreover, in the Antarctic strains, the family *Burkholderiaceae*, containing *Burkholderia* spp., accounted for >80% of the ss-HGTs from the class *Betaproteobacteria*, which suggests similar origins of the acquired ss-HGTs within the class *Betaproteobacteria* (Supplementary Figure S5B). Many of these families assigned to the class *Alphaproteobacteria* or *Betaproteobacteria* have also been reported by previous meta-16S rRNA gene analyses for moss pillars (Nakai et al., 2012), suggesting that they are likely candidate donor organisms.

Overall, HMP9 contained a high proportion of ss-HGTs obtained from more distantly related bacteria, and the two Antarctic strains have different ss-HGT origins.

It is considered that only recently acquired HGT events could be identified effectively using composition-based methods as ancient HGT genes have likely eventually evolved or ameliorated to become similar to the rest of the genome signatures (Douglas and Langille, 2019). However, we are especially interested in recently acquired HGT events in recovering Antarctic terrestrial biospheres since these biospheres in Antarctica, for example in Antarctic lake environments, had been destroyed during the glacial period and were recovered in the subsequent interglacial period (Chown et al., 2015). We would like to evaluate the recently acquired HGT events by recovering and developing the biosphere in an Antarctic lake environment after the last glacial maximum approximately 10,000 years ago (Chown et al., 2015).

### BLSOM Has Higher Detection and Classification Capabilities Than BLASTp Searches

**FIGURE 5:**
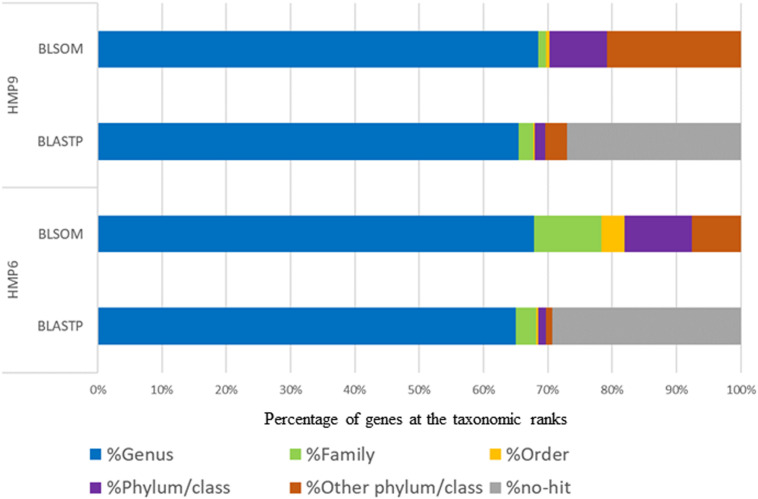
Distribution of the detected genes in Antarctic HMP6 and HMP9 strains by BLSOM and BLASTp assigned according to taxonomic rank. The horizontal bars represent proportions of the detected genes assigned to each taxonomic rank by BLSOM and BLASTp with taxonomic ranks color-coded: genus (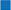), family (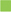), order (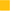), phylum/class (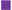), other phylum/class (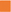), and no-hit (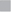).

To validate the effectiveness of the taxonomic prediction by BLSOM in terms of HGT candidate detection, we compared the taxonomic information obtained by BLSOM with that of amino acid sequence searches from BLASTp, a sequence similarity search program. Since it was difficult to define the complete positive or negative HGT gene sets in the genus *Sphingomonas*, we compared our BLSOM results with the taxonomic information obtained by BLASTp to validate our proposed method’s taxonomic prediction capacity. Figure 5 shows the genes of the Antarctic strains classified according to the taxonomic rank and obtained using these methods. The detection capability of BLSOM is higher than that of BLASTp searches with all genomic sequence segments assigned to recognized microbial origins when checking for new microbes. In contrast, ∼30% of the genes were not classified by BLASTp searches. Moreover, BLSOM can assign taxonomic information comprehensively even for microbial genomes that exhibit high novelty, which cannot be detected using sequence similarity searches.

To confirm assignment capacity, we examined the percentage of matching in comparison with taxonomic information obtained by BLSOM and BLASTp at each taxonomic rank and found that, at the genus level, the percentage of matching between BLSOM and BLASTp was 66.2% for HMP6 and 70.0% for HMP9. This low percentage of matching obtained using BLASTp was likely due to the associated difficulty in distinguishing *Sphingomonas* and *Sphingopyxis* in Genus-BLSOM, as 88.3% of *Sphingopyxis* genomic segments was mixed with *Sphingomonas* genomic segments. Therefore, the percentage of matching obtained including the two genera, *Sphingomonas* and *Sphingopyxis*, was 77.9% for HMP6 and 71.0% for HMP9. Conversely, at the family level, the percentage of matching was 81.3% for HMP6 and 72.9% for HMP9, and at the phylum/class level, the percentage of matching was 93.4% for HMP6 and 81.1% for HMP9. The percentage of matching for HMP9 was lower than HMP6 since the genes derived from the class *Betaproteobacteria* were assigned by BLSOM, and these could not be readily detected by BLASTp.

### Antarctic Strains Acquired Horizontally Transferred Genes to Adapt to the Antarctic Environment

To ascertain the types of functions acquired by the ss-HGTs of HMP6 and HMP9, we compared functional classification based on COG. The COG functional categories of the genes are shown in Supplementary Tables S4–S6.

**FIGURE 6:**
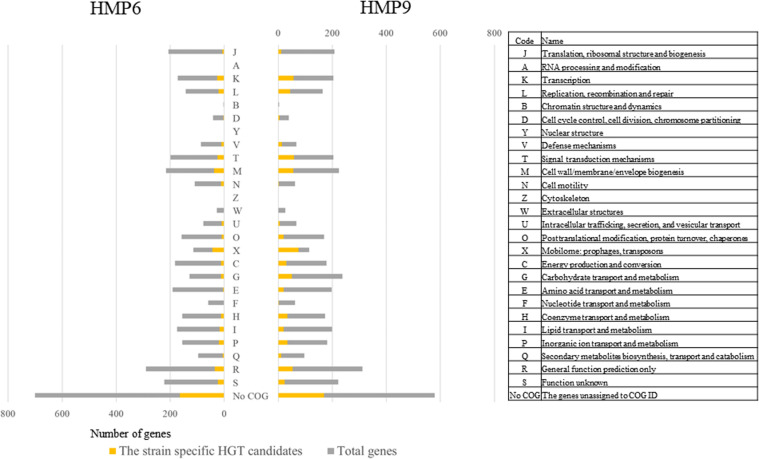
Distribution of the detected genes in Antarctic HMP6 and HMP9 strains grouped according to their COG functional categories. The COG functional codes and detailed description of COG functional categories are presented in the right-side list. Gray (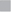): total number of total genes; orange (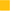): number of strain-specific HGT candidates.

Figure 6 shows the total genes and the ss-HGTs in HMP6 and HMP9 according to COG functional categories, shown on the right. Focusing on the ss-HGTs whose functions could be identified, the functional category in which the most ss-HGTs were detected was “Mobilome: prophages, transposons (X),” followed by “Cell wall/membrane/envelope biogenesis (M).” The ss-HGTs in 44 genes for HMP6 and 75 genes for HMP9 acquired through horizontal transfer belonged to “X,” which indicates that the ss-HGTs detected using BLSOM are highly reliable candidates from the viewpoint of gene functions. The functional category could not be established for 700 genes in HMP6 and 579 genes in HMP9; meanwhile, ss-HGTs was observed for 164 and 170 genes, respectively (“No COG” in Figure 6).

**FIGURE 7:**
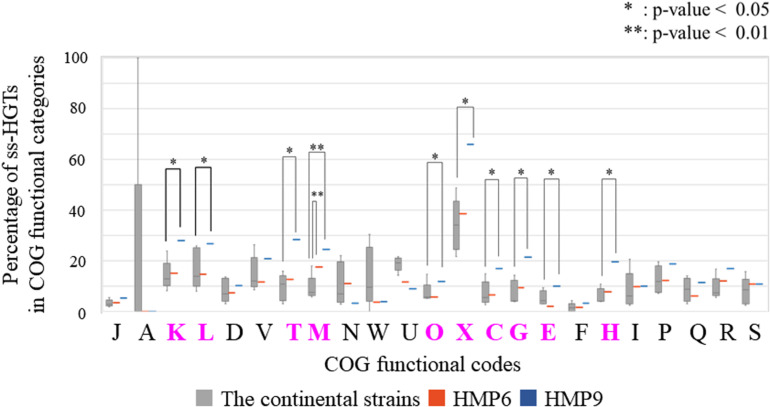
Percentage of the strain-specific HGT candidates (ss-HGTs) per COG functional category in the continental and HMP6 and HMP9 strains. Gray (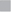): continental strains; orange bar (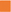): HMP6 strain; blue bar (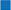): HMP9 strain. Asterisks: significant difference between continental and Antarctic strains. Pink functional categories represent those with significant differences between the continental and Antarctic HMP6 or HMP9 strains.

To analyze this comprehensively, we compared the percentages of the ss-HGTs among the genes identified in each functional category in the continental strains and in HMP6 and HMP9 (Figure 7). The proportion of ss-HGTs was significantly higher in M for HMP6 and in ten functional categories for HMP9 (K, L, T, M, O, X, C, G, E, H) compared with the continental strains. According to a gene-function analysis of interdomain HGTs from bacteria to archaea in 134 sequenced archaeal genomes, the function was unknown for 37% of the HGTs; however, the predicted gene-function categories in descending order of HGTs were E, C, G, P, T, H, M, and K (Nelson-Sathi et al., 2015). Here, the pink functional categories indicate those also detected for HMP9. Similarly, in a study conducted using a metagenomic approach on the gene functions of interdomain HGTs from bacteria to archaea, the function was unknown for 44% of the HGTs, and the predictable functions in descending order of HGTs were found to be in C, E, H, J, G, I, L, and M (Brochier-Armanet et al., 2011). Here, the pink functional categories indicate those detected for HMP9. Although the HGT predictions in these studies were based on phylogenic analyses using BLASTp, their results showed similar functional categories predicted using our BLSOM analysis.

**FIGURE 8:**
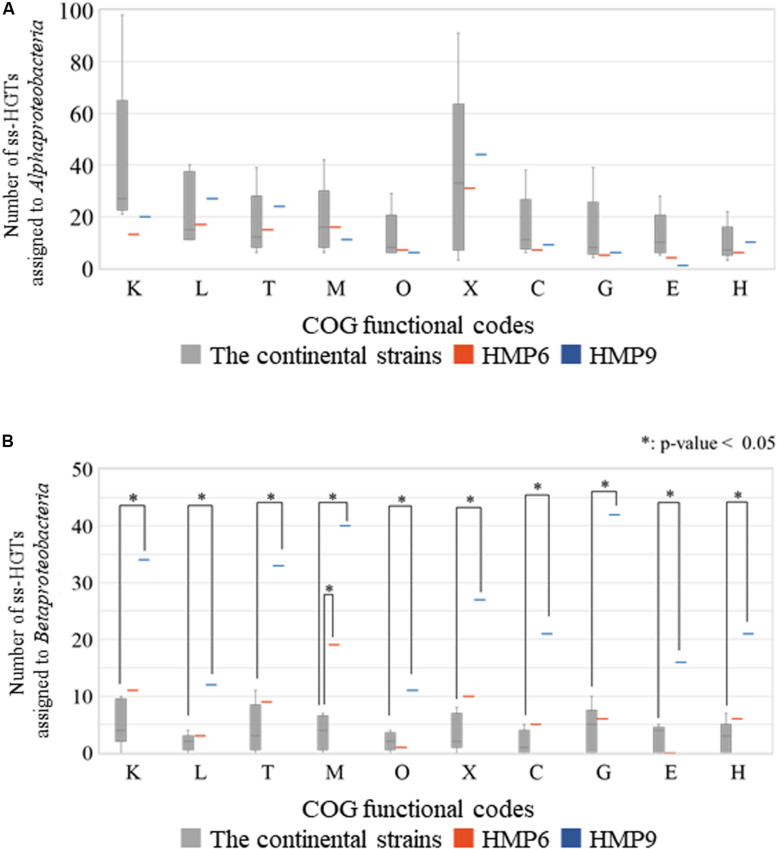
Number of strain-specific HGT candidates (ss-HGTs) assigned to **(A)** *Alphaproteobacteria* and **(B)** *Betaproteobacteria* according to their COG functional categories showing significant differences between the continental and the Antarctic HMP6 and HMP9 strains. Gray (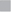): continental strains; orange bar (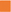): HMP6 strain; blue bar (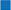): HMP9 strain. Asterisks: significant difference between continental and Antarctic strains.

To investigate the functional differences between the ss-HGTs of the Antarctic strains that were assigned to the classes *Alphaproteobacteria* and *Betaproteobacteria*, we compared the number of ss-HGTs between the continental and the Antarctic strains according to each origin among the ten functional categories in which the proportion of the ss-HGTs was predominant in the Antarctic strains (Figure 8). In the ss-HGTs derived from the class *Alphaproteobacteria*, no significant differences were found between the continental and Antarctic strains in any functional category. Meanwhile, in the ss-HGTs derived from the class *Betaproteobacteria*, a majority of the ten functional categories contained more Antarctic ss-HGTs than continental-strain ss-HGTs. Further, the number of ss-HGTs in the continental strains differed significantly in “M” for HMP6 and in ten functional categories for HMP9. Specifically, in terms of the number of ss-HGTs in HMP9, the ss-HGTs detected frequently were those belonging to “Carbohydrate transport and metabolism (G)” and “M,” which was also detected for HMP6.

Furthermore, we also constructed a 2 × 2 contingency table, calculated the odds ratio, and performed Fisher’s exact test (Supplementary Table S8). These additional tests yielded similar results as those described above.

The adaptation strategies used by psychrophiles to survive in low-temperature environments are generally suggested to include the enhancement of membrane fluidity and the biosynthesis or accumulation of compatible solutes, mainly carbohydrates related to the control of cytoplasmic osmotic pressure (De Maayer et al., 2014). For instance, HGTs in the functional categories “M” and “G” were reported in genome analysis studies of psychrophiles in the genus *Alteromonas* as part of marine bacteria of the class *Gammaproteobacteria* (Math et al., 2012), as well as of bacteria in the genus *Psychroflexus* (phylum *Bacteroidetes*) isolated from sea-ice (Feng et al., 2014). These strategies for adaptation to low-temperature environments are related to the various environmental factors such as desiccation, low nutrient availability, high or low osmotic pressure, ultraviolet radiation, and combined stresses. For example, freezing causes desiccation and results in high osmotic pressure for microbes (De Maayer et al., 2014). In the Antarctic strains HMP6 and HMP9, the functions of their genes obtained through HGTs are associated with the adaptation to environmental changes, not only to the low-temperature, but also to the conditions in Antarctic lakes, i.e., ultra-oligotrophic environment and fluctuations in sunshine and temperature during “white-night” in summer and “polar-night” in winter.

Our findings strongly suggest that these Antarctic strains might have acquired horizontally transferred genes from the class *Betaproteobacteria* for adaptation to the Antarctic environment. Moreover, HMP6 and HMP9 are suggested to harbor HGT candidates from distinct origins. However, in terms of the gene-function of the ss-HGTs derived from the class *Betaproteobacteria*, HMP6 and HMP9 had incorporated 19 and 40 genes belonging to the functional category “M,” respectively. In addition, HMP9 increased the number of ss-HGTs in ten functional categories, including “G.” Regarding the individual functions under “G,” in six genes for HMP6 and 42 genes for HMP9, the types of gene functions were nearly the same, however, the number of the ss-HGTs detected in HMP9 was frequently higher. Gene clusters consisting of these ss-HGTs including “M” or “G” were also identified. Although ss-HGTs derived from the class *Betaproteobacteria* appear to have been obtained through independent HGT events, confirming whether adjacent HGT candidates in the gene cluster are acquired in the same HGT event or via gene duplication after an HGT event, requires further investigation through BBH relationships and phylogenetic tree analysis. It is, however, imperative that these further investigations take into account taxonomic assignment and construction of gene families, when conducting best BLAST hit analysis (Dick et al., 2017). Thus, the Antarctic strains might have acquired some of the gene functions required for adaptation to the Antarctic environment from the class *Betaproteobacteria*, and multiple HGT acquisition mechanisms might underlie these HGT events.

### Amino Acid Frequency Analysis to Examine Relationship Between HGT Candidates and Adaptation to Antarctic Environment

Adaptations to low temperatures and oligotrophic environments are examples of special adaptations found in the extreme environment of the Antarctic (Feng et al., 2014). Low-temperature adaptation is necessary for psychrophilic bacteria to thrive at low temperatures, wherein the chemical reaction rate is low, and for stable mass transport and metabolism reactions to occur. Specifically, low-temperature adaptation is influenced by modifications of coding for lysine and arginine in cold-adapted genes that exhibit this tendency, and it has been reported that the low-temperature adaptation of the affected proteins can be examined by investigating their low arginine content and Arg/(Arg + Lys) ratio (Russell, 2000).

**FIGURE 9:**
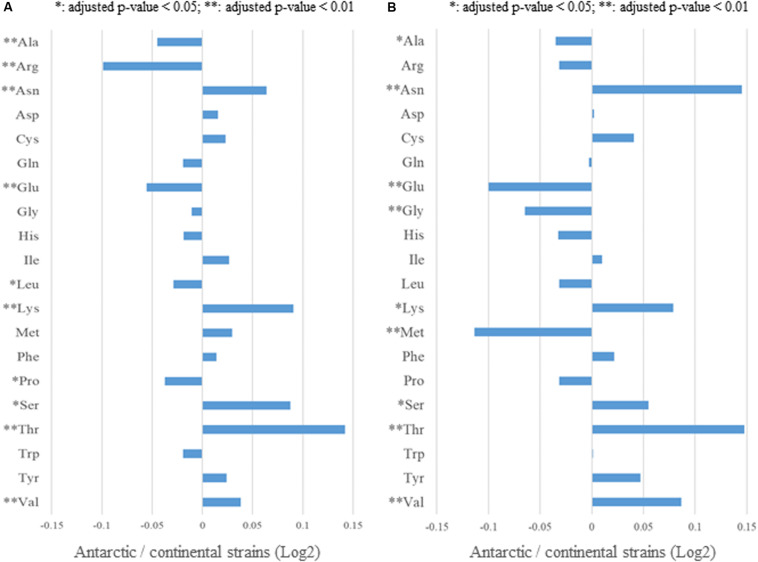
Comparison of amino acid usage between the Antarctic and continental strains. **(A)** Ratio of amino acid frequencies in the housekeeping genes of the Antarctic and continental strains. Asterisks: significant difference between Antarctic and continental strains. **(B)** Ratio of amino acid frequencies in the strain-specific HGT candidates of the Antarctic and continental strains.

To examine the relationship between HGT candidates and adaptation to the Antarctic environment, we focused on the amino acid frequency of proteins of the Antarctic and continental strains. First, we searched for significant differences between the Antarctic and continental strains, in terms of the amino acid sequences of proteins encoded by 856 housekeeping genes, whose conservation was determined by BBH analysis and reported complete genomic sequences and optimal growth temperatures (Figure 2 and Supplementary Table S2). The ratio of the amino acid frequencies of housekeeping genes between the Antarctic and continental strains are shown in Figure 9A, whereas the actual numbers, means, and significant differences are listed in Supplementary Table S9. In the Antarctic strains, increased frequencies for five amino acids (Asn, Lys, Ser, Thr, Val), and decreased frequencies for five (Ala, Arg, Glu, Leu, Pro), were observed for housekeeping genes. Secondly, we identified significant differences between the Antarctic and continental strains in terms of the amino acid frequencies of the ss-HGTs, the ratio of which are shown in Figure 9B, and the actual numbers, means, and significant differences are listed in Supplementary Table S10. The ss-HGTs showed a similar tendency as those of the housekeeping genes, save for two amino acids (Met and Trp) that indicated opposite tendencies (Figures 9A,B and Supplementary Tables S9, S10). Our findings regarding the amino acid frequencies of housekeeping genes and ss-HGTs between the Antarctic and continental strains are consistent with those of a previous study that reported low arginine content and an Arg/(Arg + Lys) ratio in cold-adapted genes (Russell, 2000).

We also examined the amino acid frequencies of housekeeping genes compared to each clade of Antarctic strain HMP6 and HMP9 and found that HMP6 formed a clade with *S. psychrolutea* MDB1-A (Figure 2). However, as the genomic sequence data has not been previously reported for MDB1-A we instead analyzed *Sphingomonas* sp. AAP5 and *Sphingomonas panacis* DCY99 with HMP6 (Supplementary Figure S2). Although the complete genome sequences have been reported for these *Sphingomonas* strains, their optimal growth temperatures have not (Supplementary Table S7). Our results show that HMP9 formed a clade with *S. aerolata* NW12 and *S. ginsenosidivorax* KHI67 (Figure 2), however, this genome sequencing data was not complete but rather was draft data containing sequence gaps (Supplementary Table S7). The distribution of amino acid frequencies in comparison with the strains in each HMP6 or HMP9 clade and the continental strains are shown in Supplementary Figures S6, S7, respectively. It was suggested that the amino acid frequencies of housekeeping genes in these strains in each clade showed a similar tendency in Arg, Glu, Ser, and Thr with the Antarctic strain HMP6 and HMP9. From these results, it may be suggested that the Antarctic strains HMP6 and HMP9 evolved independently in each clade and that their low-temperature adaptations occurred convergently (Supplementary Figure S2).

Regarding the properties of the amino acids, Ser and Thr are hydrophilic, whereas Ala and Leu are hydrophobic. Moreover, Arg can form more salt bridges and hydrogen bonds with surrounding residues through guanidino groups than Lys, thus helping to stabilize the proteins (Russell, 2000). Psychrophilic strains of the genus *Shewanella* and *Gammaproteobacteria* were found to have decreased G + C content and proteome Ala, Pro, and Arg contents (Zhao et al., 2010). Since the Antarctic strains and psychrophilic bacteria exhibit similar characteristics in terms of amino acid frequency, the Antarctic strains may have adapted to low-temperature environments by increasing the flexibility of their protein structures and decreasing the activation energy of a chemical reaction (Russell, 2000; Marx et al., 2007).

**FIGURE 10:**
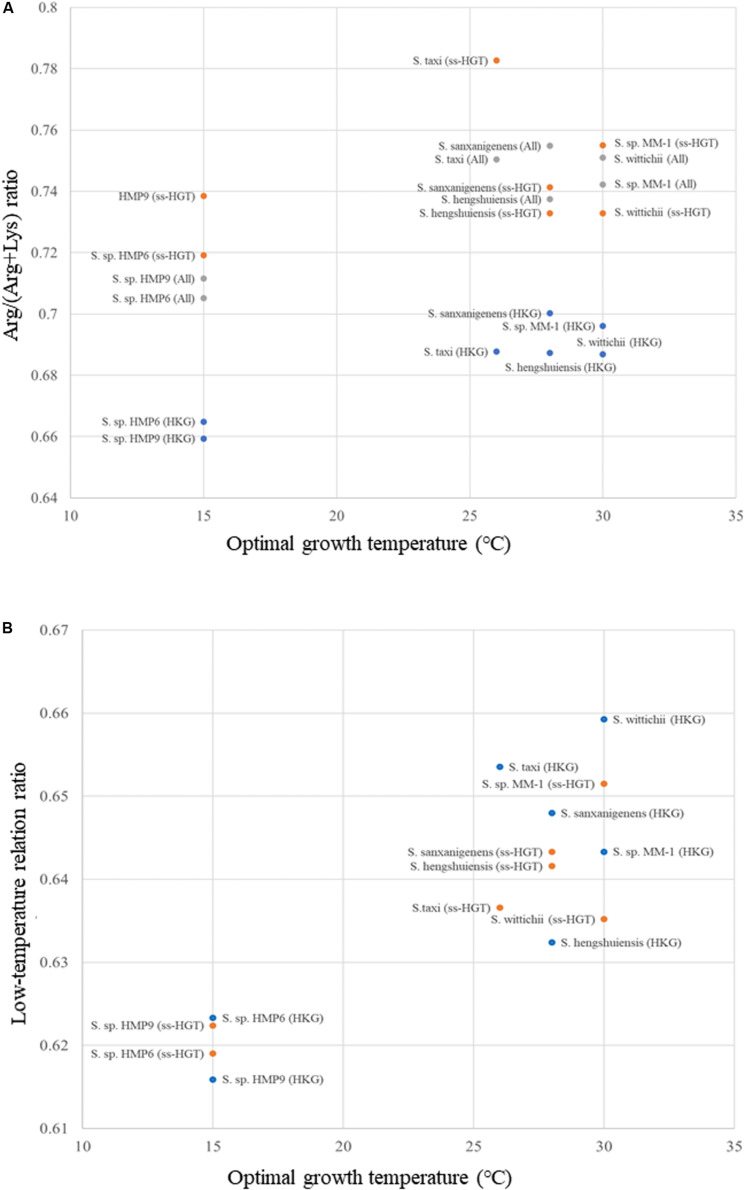
Scatter plot of amino acid ratio versus optimal growth temperature of *Sphingomonas* strains. **(A)** Arg/(Arg + Lys) ratio. Blue: housekeeping genes (HKG); orange: strain-specific HGT candidates (ss-HGT); gray: all genes (All). **(B)** Low-temperature relation ratio. Blue: housekeeping genes (HKG); orange: strain-specific HGT candidates.

To confirm the low-temperature adaptation, we investigated the Arg/(Arg + Lys) ratio, and its relationship with optimal growth temperature, which is displayed in Figure 10A. Based on this relationship (Zeldovich et al., 2007), we selected Lys, Ser, Thr, and Val as amino acids that were increased in the Antarctic strains, whereas Ala, Arg, Glu, and Leu were those decreased in the Antarctic strains. From this we developed a formula (see section “Materials and Methods”) defined as the low-temperature relation ratio for Antarctic *Sphingomonas* strains. The relationship between the optimal growth temperature and the low-temperature relation ratio is plotted in Figure 10B. In terms of the housekeeping genes (blue dots in Figures 10A,B), the continental strains and the Antarctic strains showed high and low values on the low-temperature relation ratio, respectively, suggesting that changes in amino acid frequency for low-temperature adaptation occurred in the Antarctic strains. Conversely, in terms of the ss-HGTs (orange dots), the ratios for the ss-HGTs of the Antarctic strains were as low as those of the housekeeping genes of the Antarctic strains, suggesting that changes in amino acid frequency for low-temperature adaptation occurred in not only the housekeeping genes but also in the ss-HGTs, which indicates these changes potentially occurred in the entire genome.

We also compared the low-temperature relation ratios for individual genes (Supplementary Figure S8). The low-temperature relation ratios of both the housekeeping genes and ss-HGTs were spread widely, however, the spread ranges were similar among the *Sphingomonas* strains. The changes in the amino acid frequencies for low-temperature adaptation could potentially occur in the entire genome based on the results of the housekeeping genes. The relationship between the HGT genes and changes in the amino acid frequency after the HGT events is an important issue to be considered for further study as it could serve as a key factor in the elucidation of the low-temperature adaptation process in Antarctic bacteria. Therefore, we conclude that the ss-HGTs may be involved in the adaptation of genes to the Antarctic environment, including that to low temperatures, although the origins of the ss-HGTs of the two Antarctic strains are different.

## Conclusion

The method we have developed for detecting HGT candidates employs BLSOM, an algorithm based on oligonucleotide usage that does not depend on sequence similarity. In other words, HGT candidates are identified and their origin is assigned with high efficiency based solely on references to oligonucleotide usage. In our proposed workflow, although BLSOM was constructed using a degenerate tetranucleotide composition and 5-kb window featuring 1-kb step, similar results can also be obtained with different conditions, for example using segment length or oligonucleotides, as shown by our previous studies (Abe et al., 2003; Iwasaki et al., 2013). Our method is applicable to both complete and draft genomes.

From the ss-HGT candidates we detected in two Antarctic strains of *Sphingomonas*, HMP6 and HMP9, through BLSOM and BBH analysis, we found that HMP6 harbored a higher number of genes acquired through horizontal transfer from the class *Alphaproteobacteria*, which is closely related to *Sphingomonas* spp., whereas HMP9 harbored a higher number of genes acquired from the class *Betaproteobacteria*, which is relatively more distantly related to *Sphingomonas* spp. This finding clearly shows an origin difference in the horizontally transmitted genes found in the two Antarctic strains, thus suggesting that horizontally transferred genes were obtained through multiple acquisition processes occurring in the same Antarctic environment.

In terms of gene functions, both Antarctic strains have obtained genes related to cell walls or cell membranes mainly from the class *Betaproteobacteria*. Particularly, HMP9 has acquired ten gene functions, including those involved in carbohydrate metabolism from the class *Betaproteobacteria*. Moreover, in relation to adaptability to the Antarctic environment, these ss-HGT candidates exhibited changes in amino acid frequency suited for low-temperature adaptation, and these changes have occurred throughout the genome. Similarly, this frequency change was observed in ss-HGTs. Thus, it is highly likely that the ss-HGTs are related to adaptation in the Antarctic environment, including that in low-temperature. The origins and the genetic functions of the horizontally transferred genes suggest that multiple pathways and strategies underlie adaptation to the Antarctic environment.

Our results indicate that our proposed BLSOM analysis could serve as a powerful tool for detecting HGT candidates and their origins in entire genomes. Our findings not only the enhance current understanding of how the two Antarctic *Sphingomonas* strains have adapted to their living environment, but also provide more general insights into the adaptation process in terms of amino acid composition changes that occur via HGT.

Generally, the compositional method primarily identifies recent transfers, while the phylogenetic method is more suitable for the detections of older events (Tamames and Moya, 2008). We have recently developed a new hierarchical BLSOM according to data class, such as phylotype (Kikuchi et al., 2015), and will aim to develop a new powerful method that makes use of the characteristics of both the compositional and phylogenetic methods to detect recent and older HGTs efficiently. This platform will allow for HGT acquisition and adaptation processes combined with the amino acid frequency analysis in future studies.

The large-scale destruction and reconstruction of ecosystems are presumed to have occurred in the glacial and interglacial cycles in Antarctica (Chown et al., 2015), and it is considered that various adaptation strategies have been tested in each organism or biosphere during this process. Functional genes for cellular process and energy metabolism that were acquired by horizontal transfer are considered to have been affected by changes in the amino acid frequency of the proteins that these genes encode, and Antarctic bacteria have acquired genes from other bacteria already adapted to low-temperature environments; therefore, these genes and the bacteria have shown high environmental adaptability even under conditions in which genetic resources are limited, such as in Antarctica. The adaptability has occurred convergently as independent evolutionary strategies in each Antarctic strain.

## Data Availability Statement

The datasets generated for this study can be found in the DNA Data Bank of Japan under accession numbers AP022672 for *Sphingomonas* sp. HMP6, AP022673 for *Sphingomonas* sp. HMP9 and AP022674 for its plasmid named pHMP9.

## Author Contributions

TA and TB designed the research. TA, YA, and TB performed the research. AT and HN contributed new materials. TA, YA, AT, and TB analyzed the data. All authors contributed to the article and approved the submitted version.

## Conflict of Interest

The authors declare that the research was conducted in the absence of any commercial or financial relationships that could be construed as a potential conflict of interest.

## Footnotes

1 http://bioinfo.ie.niigata-u.ac.jp/?BLSOM

2 https://ftp.ncbi.nlm.nih.gov/genbank/

3 ftp://ftp.ddbj.nig.ac.jp/ddbj_database/wgs/

4 http://bioinfo.ie.niigata-u.ac.jp/?PEMS4HGT

5 https://www.ncbi.nlm.nih.gov/refseq/targetedloci/
