# Supplementary material for: Batch-Learning Self-Organizing Map Identifies Horizontal Gene Transfer Candidates and Their Origins in Entire Genomes
Source: Front Microbiol. 2020 Jul 3;11:1486. doi: 10.3389/fmicb.2020.01486 (PMC7350273; doi:10.3389/fmicb.2020.01486)
Supplement: Supplementary file 11 [file Image_1.pdf]

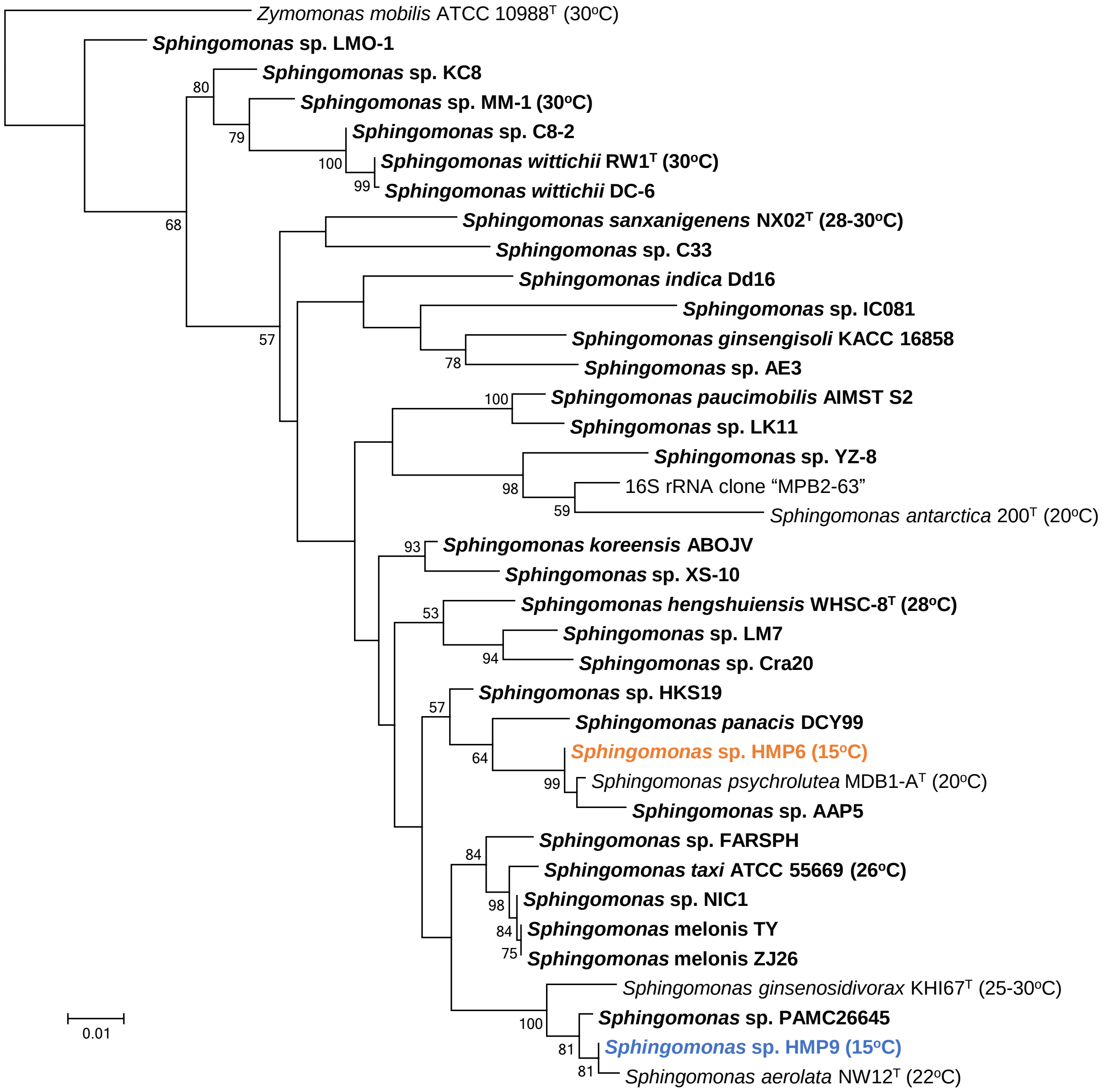

Supplementary Figure 1. Phylogenetic tree based on 16S rRNA sequence data of 36 *Sphingomonas* strains, constructed using the maximum-likelihood method under the Tamura-Nei model. *Zymomonas mobilis* ATCC 10988 was used as an outgroup. Species names in bold lettering: genome-sequenced strains; values in parentheses: growth temperatures; species names with superscript “T”: type strains; numbers next to branches: bootstrap percentage values for 1000 replicates. Values inside parentheses are temperatures of optimum growth. Orange and blue text pepresents the Antarctic HMP6 and HMP9 strains, respectively.
