# Supplementary material for: Batch-Learning Self-Organizing Map Identifies Horizontal Gene Transfer Candidates and Their Origins in Entire Genomes
Source: Front Microbiol. 2020 Jul 3;11:1486. doi: 10.3389/fmicb.2020.01486 (PMC7350273; doi:10.3389/fmicb.2020.01486)
Supplement: Supplementary file 12 [file Image_2.pdf]

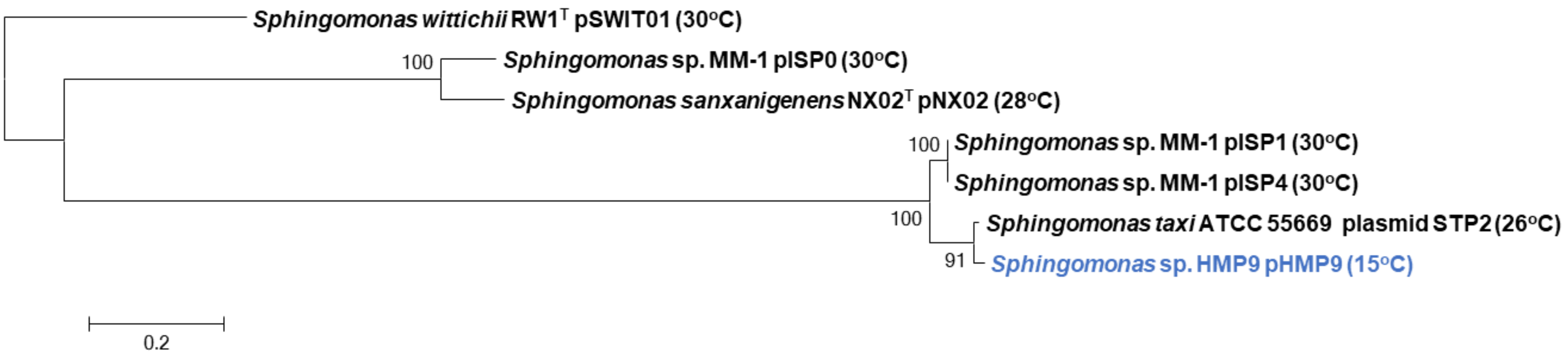

Supplementary Figure 2. Phylogenetic tree based on RepB gene sequence data of 7 *Sphingomonas* plasmids, constructed using the maximum-likelihood method under the Tamura-Nei model. Species names in bold lettering: genome-sequenced plasmids; values in parentheses: growth temperatures; plasmids names with superscript “T”: type strains; numbers next to branches: bootstrap percentage values for 1000 replicates. Values inside parentheses are temperatures of optimum growth. Blue text represents *Sphingomonas* sp. HMP9 pHMP9 plasmid.
