# Supplementary material for: Batch-Learning Self-Organizing Map Identifies Horizontal Gene Transfer Candidates and Their Origins in Entire Genomes
Source: Front Microbiol. 2020 Jul 3;11:1486. doi: 10.3389/fmicb.2020.01486 (PMC7350273; doi:10.3389/fmicb.2020.01486)
Supplement: Supplementary file 13 [file Image_3.pdf]

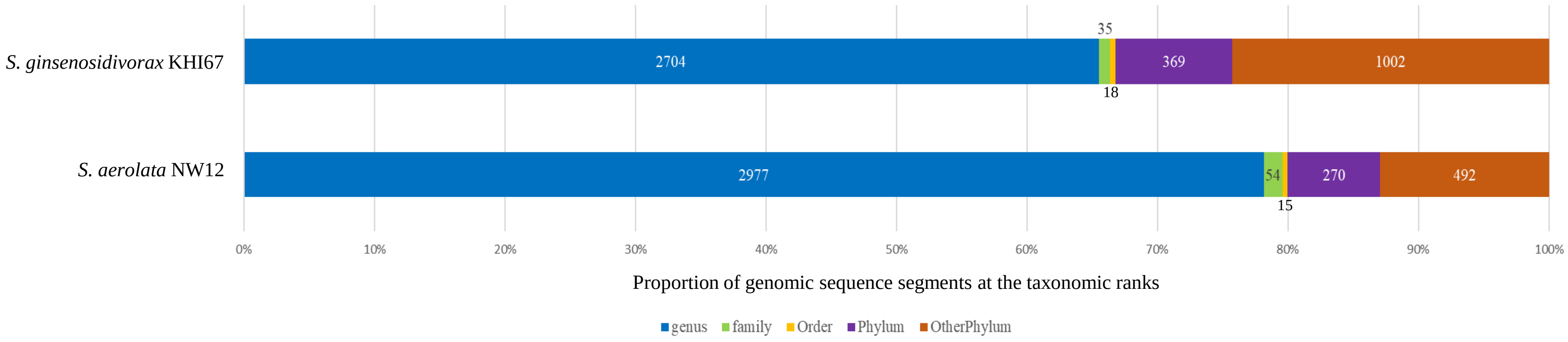

Supplementary Figure 3.
Proportion of genomic sequence segments in draft genomes *S. ginsenosidivorax* KHI67 and *S. aerolata* NW12. The number in each taxonomic rank is the number of genomic segment sequences assigned to the taxonomic rank. Horizontal bars: proportion of genomic segments assigned to each taxonomy level by BLSOM; taxonomy levels are color-coded thus: genus ( ), family ( ), order ( ), phylum/class ( ), and other phylum/class ( ).
