# Supplementary material for: Batch-Learning Self-Organizing Map Identifies Horizontal Gene Transfer Candidates and Their Origins in Entire Genomes
Source: Front Microbiol. 2020 Jul 3;11:1486. doi: 10.3389/fmicb.2020.01486 (PMC7350273; doi:10.3389/fmicb.2020.01486)
Supplement: Supplementary file 14 [file Image_4.pdf]

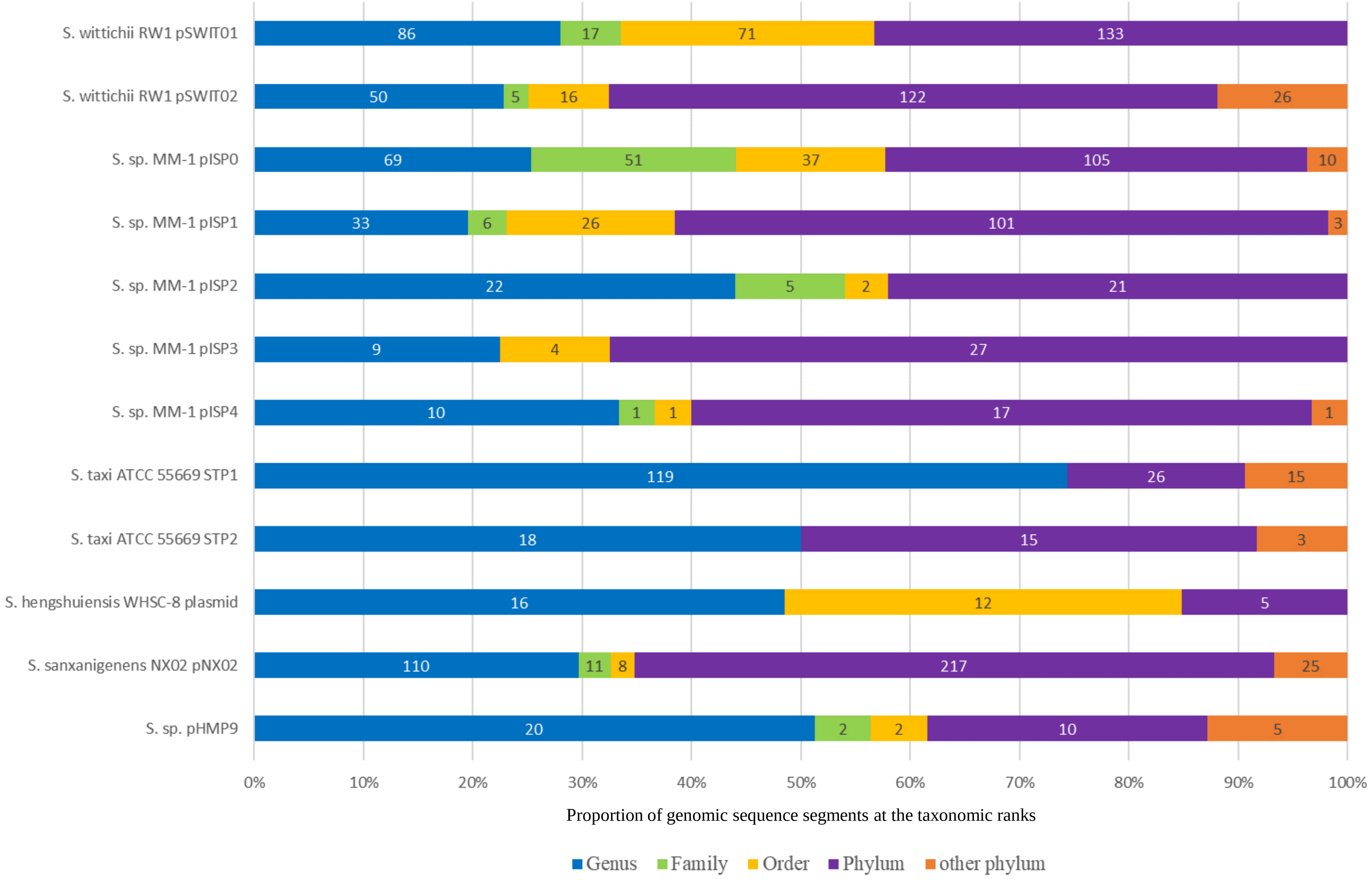

Supplementary Figure 4.
Proportion of genomic sequence segments in the *Sphingomonas* plasmid genomes. The number in each taxonomic rank is the number of genomic segment sequences assigned to the taxonomic rank. Horizontal bars: proportion of genomic segments assigned to each taxonomy level by BLSOM; taxonomy levels are color-coded thus: genus (■), family (■), order (■), phylum/class (■), and other phylum/class (■).
