# Supplementary material for: Batch-Learning Self-Organizing Map Identifies Horizontal Gene Transfer Candidates and Their Origins in Entire Genomes
Source: Front Microbiol. 2020 Jul 3;11:1486. doi: 10.3389/fmicb.2020.01486 (PMC7350273; doi:10.3389/fmicb.2020.01486)
Supplement: Supplementary file 15 [file Image_5.pdf]

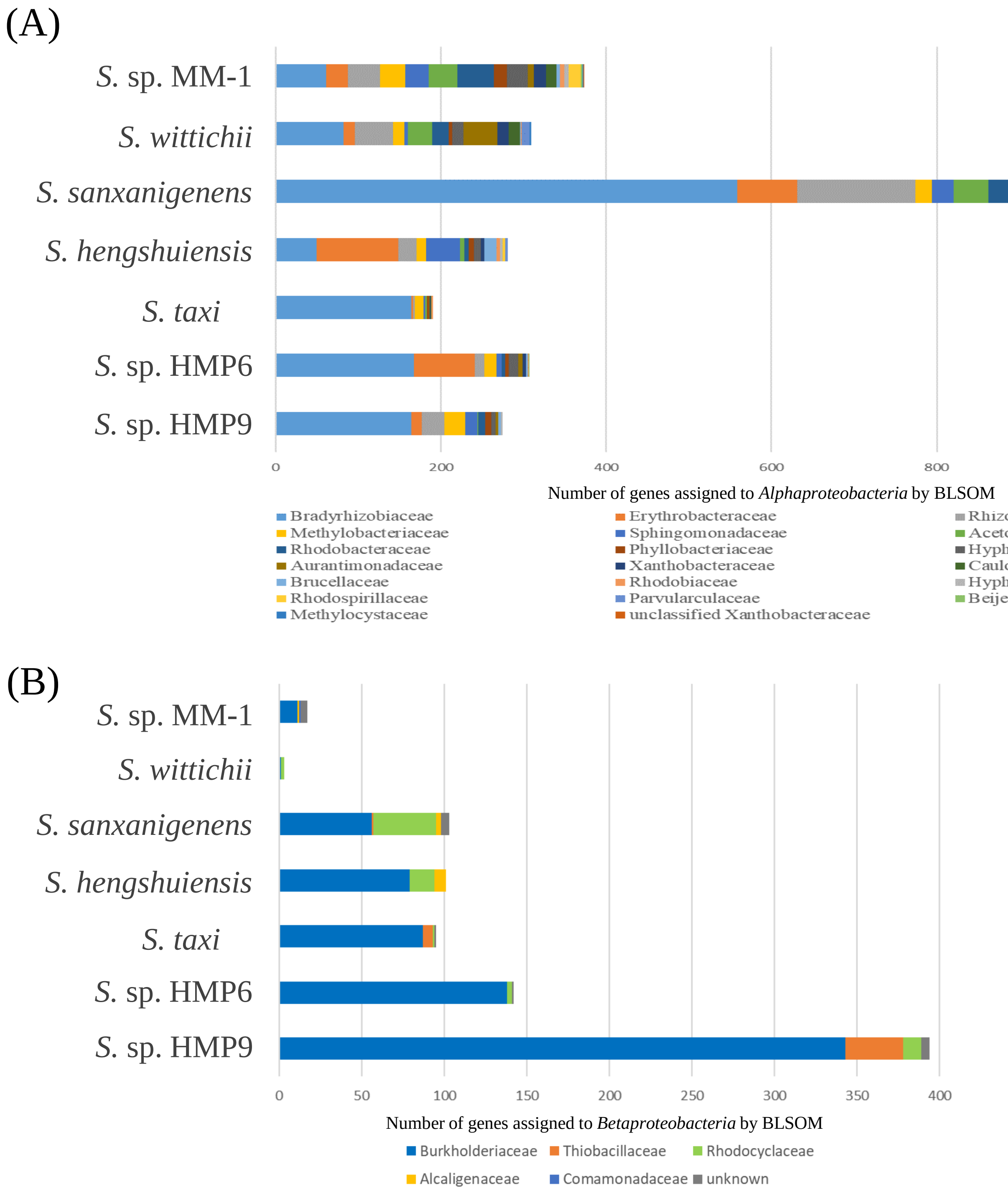

Supplementary Figure 5 Comparison of number of genes assigned to *Alphaproteobacteria* (A) and *Betaproteobacteria* (B) at family level. Family names and color codes are shown at the bottom of each panel.
