# Supplementary material for: Batch-Learning Self-Organizing Map Identifies Horizontal Gene Transfer Candidates and Their Origins in Entire Genomes
Source: Front Microbiol. 2020 Jul 3;11:1486. doi: 10.3389/fmicb.2020.01486 (PMC7350273; doi:10.3389/fmicb.2020.01486)
Supplement: Supplementary file 16 [file Image_6.pdf]

(A)

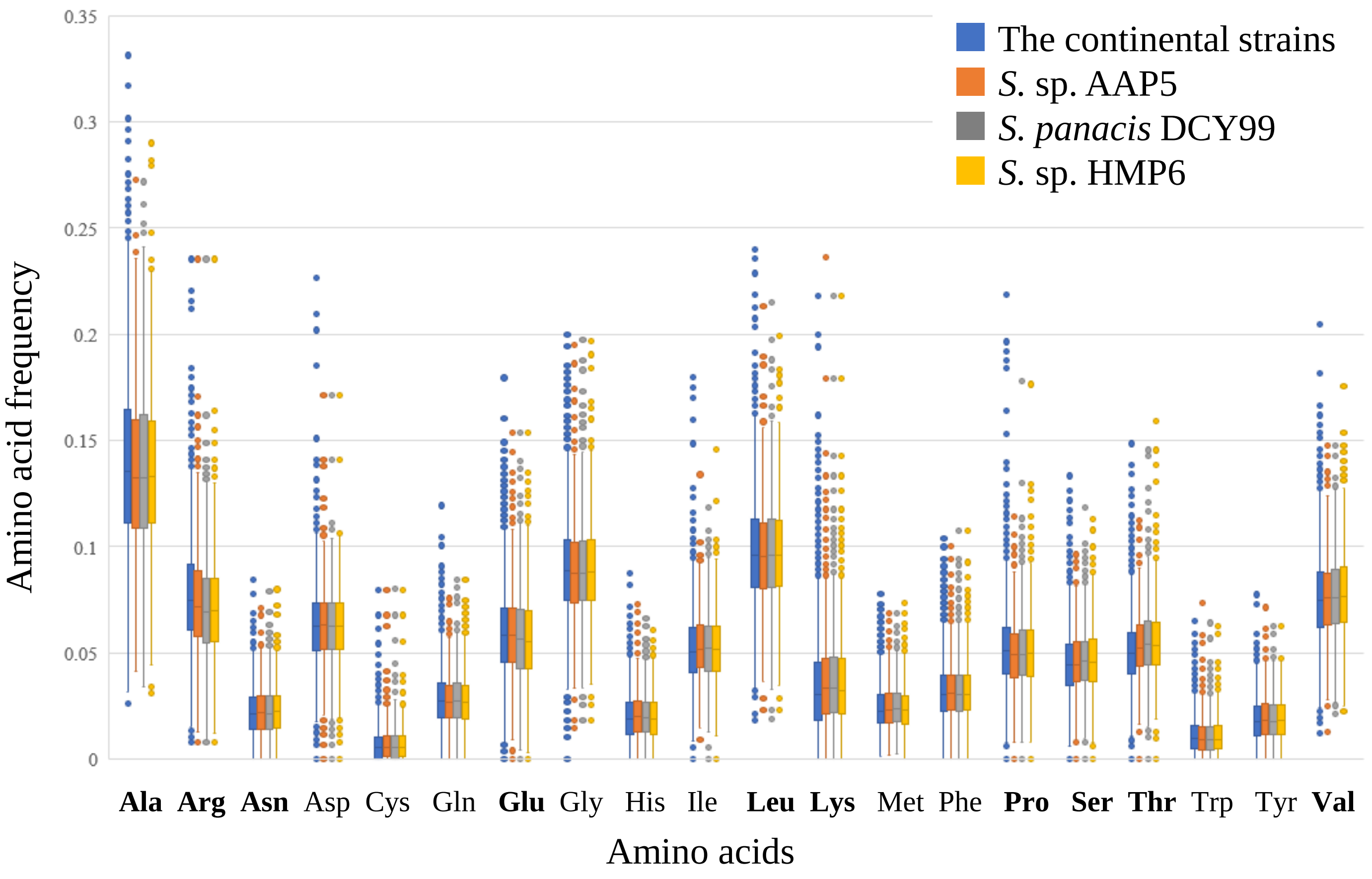

(B)

	Mean Freq.				Significance test result (adjusted p-value) The continental strains vs		
Amino Acids	The continental strains*1	*S. panacis* DCY99	*S*. sp. AAP5	*S*. sp HMP6	*S. panacis* DCY99	*S*. sp. AAP5	*S*. sp HMP6
**Ala**	0.1386	0.1351	0.1360	0.1360	0.12	0.15	0.15
**Arg**	0.0767	0.0739	0.0710	0.0716	0.0094	5.80E-09	8.50E-08
**Asn**	0.0224	0.0230	0.0227	0.0230	0.44	0.72	0.44
Asp	0.0619	0.0626	0.0616	0.0618	0.78	0.86	0.86
Cys	0.0072	0.0074	0.0074	0.0074	0.94	0.94	0.94
Gln	0.0288	0.0279	0.0285	0.0279	0.19	0.6	0.19
**Glu**	0.0593	0.0598	0.0577	0.0570	0.54	0.11	0.032
Gly	0.0893	0.0880	0.0888	0.0889	0.76	0.76	0.76
His	0.0202	0.0210	0.0202	0.0197	0.26	0.96	0.34
Ile	0.0521	0.0535	0.0526	0.0531	0.15	0.57	0.28
**Leu**	0.0979	0.0967	0.0976	0.0974	0.86	0.86	0.86
**Lys**	0.0355	0.0378	0.0379	0.0373	0.06	0.042	0.076
Met	0.0245	0.0248	0.0251	0.0245	0.75	0.52	0.84
Phe	0.0321	0.0326	0.0324	0.0325	0.94	0.94	0.94
**Pro**	0.0521	0.0497	0.0507	0.0510	0.0034	0.143	0.2
**Ser**	0.0450	0.0459	0.0471	0.0476	0.16	1.10E-03	9.30E-05
**Thr**	0.0504	0.0537	0.0553	0.0551	2.30E-07	4.90E-13	4.90E-13
Trp	0.0114	0.0110	0.0111	0.0113	0.85	0.85	0.85
Tyr	0.0186	0.0193	0.0189	0.0189	0.66	0.66	0.66
**Val**	0.0761	0.0765	0.0772	0.0777	0.62	0.47	0.24

*1. The continental strains are *S*. taxi ATCC 55669, *S. hengshuiensis* WHSC-8, *S. sanxanigenens* NX02, *S. wittichii* RW1 and *S*. sp. MM-1.

Supplementary Figure 6. Distribution of amino acid frequencies in housekeeping genes. (A) Boxplot of amino acid frequencies in housekeeping genes of HMP6 and closely related strains. Bold amino acids: Amino acids with significant differences in Figure 9A. (B) The mean frequencies of amino acids and adjusted p-values of significance tests. The *t* tests for the continental strains vs HMP6 and closely related strains were performed for each amino acid. Yellow column: significant difference. Bold amino acids: Amino acids with significant differences in Figure 9A.
