# Supplementary material for: Batch-Learning Self-Organizing Map Identifies Horizontal Gene Transfer Candidates and Their Origins in Entire Genomes
Source: Front Microbiol. 2020 Jul 3;11:1486. doi: 10.3389/fmicb.2020.01486 (PMC7350273; doi:10.3389/fmicb.2020.01486)
Supplement: Supplementary file 18 [file Image_8.pdf]

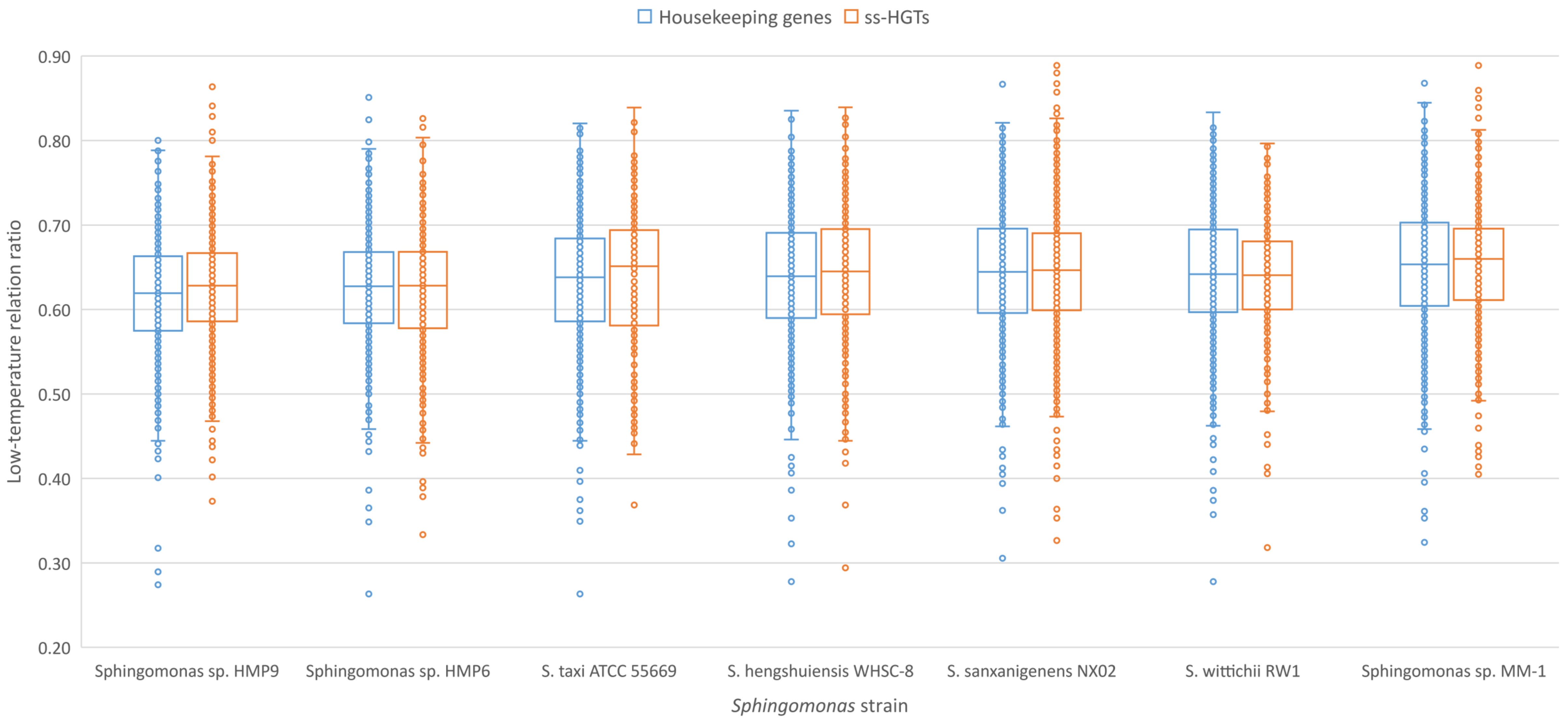

Supplementary Figure 8. Boxplot of individual low-temperature relation ratio values of the housekeeping genes and ss-HGTs in each *Sphingomonas* strain. Clear blue: housekeeping genes; clear orange: ss-HGTs
